# Clinical immunotherapy in glioma: current concepts, challenges, and future perspectives

**DOI:** 10.3389/fimmu.2024.1476436

**Published:** 2024-11-01

**Authors:** Jun Liu, Jingjian Peng, Jian Jiang, Yanhui Liu

**Affiliations:** ^1^ Department of Neurosurgery, West China Hospital, Sichuan University, Chengdu, Sichuan, China; ^2^ Department of Neurosurgery, Jiujiang No. 1 People’s Hospital, Jiujiang, China

**Keywords:** immunotherapy, gliomas, tumor vaccines, immune checkpoint inhibitors, CAR-T, oncolytic virotherapy

## Abstract

Glioma is one of the common tumors in the central nervous system, and its treatment methods (surgery, radiotherapy, and chemotherapy) lack specificity and have a poor prognosis. With the development of immunology, cell biology, and genomics, tumor immunotherapy has ushered in a new era of tumor therapy, achieving significant results in other invasive cancers such as advanced melanoma and advanced non-small cell lung cancer. Currently, the clinical trials of immunotherapy in glioma are also progressing rapidly. Here, this review summarizes promising immunotherapy methods in recent years, reviews the current status of clinical trials, and discusses the challenges and prospects of glioma immunotherapy.

## Introduction

**Figure 1:**
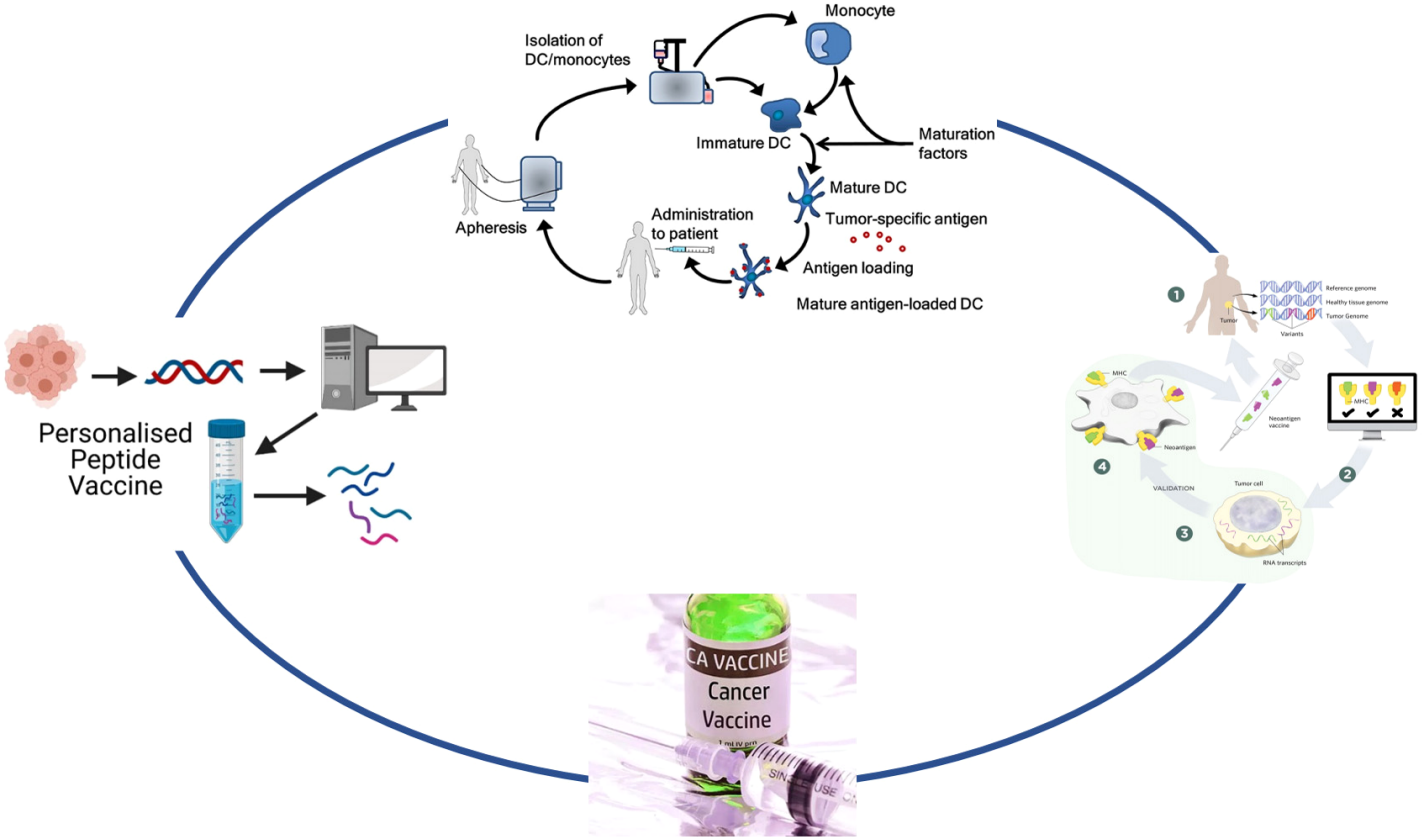
Strategies to overcome gliomas. The diagram illustrates various immunotherapy strategies for targeting and eliminating cancer cells. On the left, cancer vaccines stimulate an immune response, while immune checkpoint inhibitors like Ipilimumab, Nivolumab, Pembrolizumab, and Cemiplimab block the PD-1 pathway to activate T-cells. PD-L1 inhibitors such as Atezolizumab, Durvalumab, and Avelumab further promote the killing of cancer cells by preventing cancer cells from evading immune detection. CAR-T cell therapy is depicted, showing how T-cells are engineered with a chimeric antigen receptor (CAR) to directly attack tumor cells. Oncolytic viruses are also represented, illustrating their role in selectively infecting and destroying cancer cells.

Glioma refers to a tumor originating from glial cells and is the most common primary tumor in the central nervous system (1). Glioma, with an incidence of 6.6/100,000 of which about half were glioblastomas (1), accounts for almost 90% of all malignant brain and other central nervous system tumors (2). The incidence of glioma increases with age (3), and the incidence of glioblastoma was highest in males, persons aged more than 65 years, and non-Hispanic White (2). The symptoms of glioma depend on their location, size, type, and growth rate. Glioma treatment usually begins with surgery and is followed by radiation therapy, chemotherapy, and targeted therapy. However, most patients are not sensitive to traditional treatment and have a poor prognosis. Available treatment options include second-line surgery, radiotherapy, alkylating agent chemotherapy, and bevacizumab therapy. Unfortunately, once progression or recurrence occurs, the median overall survival (OS) is only 6 to 9 months. Therefore, there is an urgent need for new therapeutic strategies to treat recurrent GBM (4). With the advancement of immunology, cell biology, and molecular biology, tumor immunotherapy has ushered in a new era of cancer treatment (5). Immunotherapy as a new treatment method may be beneficial for delaying glioma recurrence and improving the therapeutic effect of glioma. Recently, immunotherapy has achieved some exciting and encouraging results even though there are still many challenges in practical clinical applications. A deeper understanding of the biology and immune microenvironment, along with the development of new therapeutic combinations, may potentially change the current challenges faced by immunotherapy in GBM. We provide a review of the current status and new developments in immunotherapy, including tumor vaccines, immune checkpoint inhibitors, chimeric antigen receptor T cells (CAR-T), and oncolytic virotherapy, for glioma (**Figure 1**).

## Tumor vaccines

**Figure 2:**
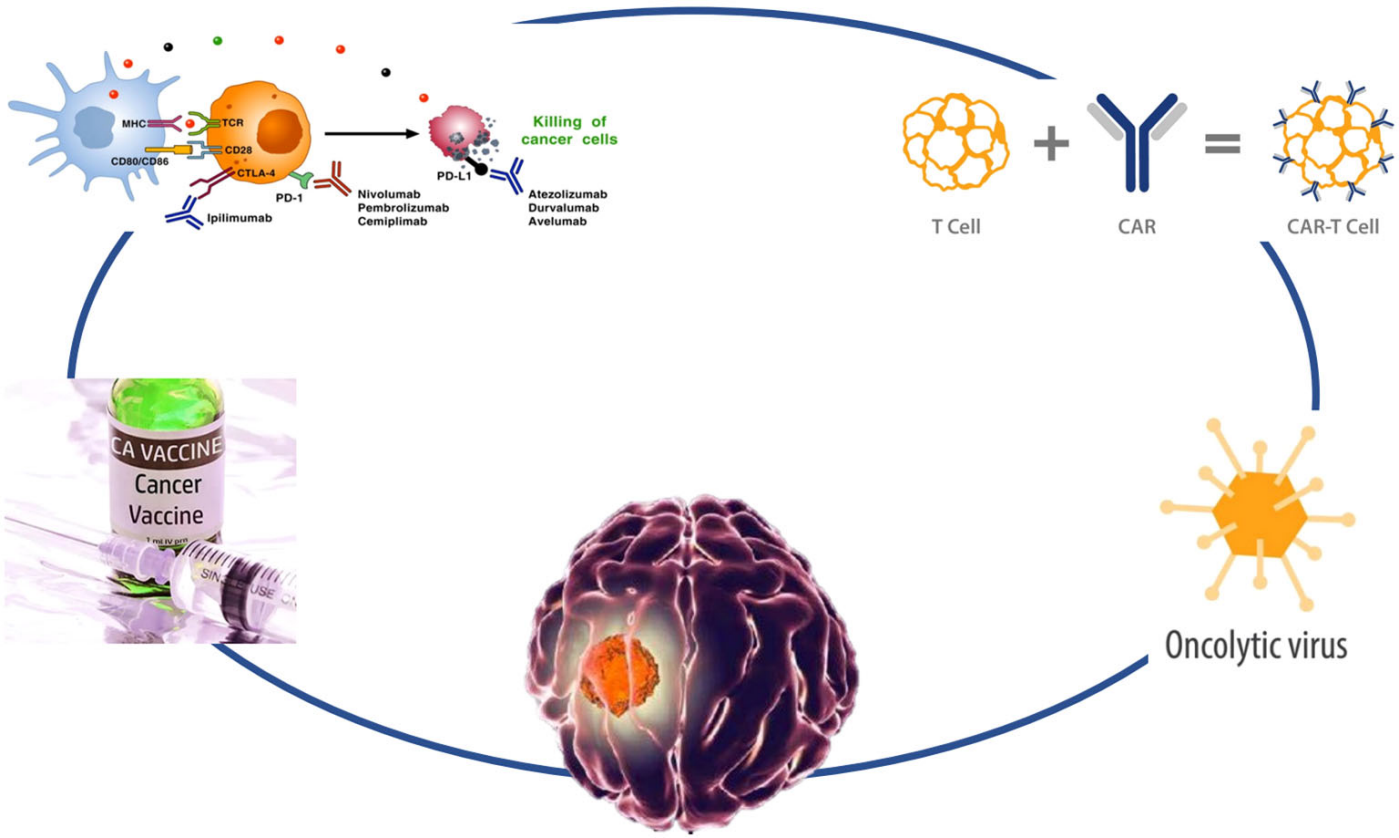
Classification of tumor vaccines. This diagram illustrates two personalized immunotherapy approaches for cancer: peptide vaccines and dendritic cell (DC) therapy. On the left, the process of creating a personalized peptide vaccine begins with the identification of tumor-specific antigens from the patient’s tumor cells, followed by the generation of a peptide vaccine tailored to the patient. On the right, dendritic cell therapy involves isolating monocytes from the patient, differentiating them into immature DCs, and loading them with tumor-specific antigens. These mature antigen-loaded DCs are then reintroduced into the patient to stimulate an immune response.

The use of vaccines to treat confirmed malignant tumors can be traced back to 1910s and 1950s (6). Tumor vaccines can utilize the adaptive immune system to produce tumor-specific antibodies and thereby exert anti-tumor effects (7), mainly divided into peptide vaccine, dendritic cell vaccine, and tumor neoantigen vaccine (8) (**Figure 2**).

## Peptide vaccine

Peptide vaccine is a vaccine made by artificially synthesizing protective short peptides in the amino acid sequence of natural proteins, connecting them with carriers, and adding adjuvants. A phase I clinical study conducted in Japan indicated that WT1 peptide vaccine induced WT1-specific CD8+ T cells and CD4+ T cells and was tolerable in patients with WT1+ malignant glioma (9), and the phase II clinical trial further showed WT1 peptide vaccine was safe and produced a clinical response in patients with WT1/HLA-A*2402+ recurrent glioblastoma multiforme (10). During the administration of the WT1 peptide vaccine, the maintenance of WT1 expression in tumor cells is significantly associated with a longer progression free and overall survival (11). ACT IV, a randomized, double-blind, phase 3 study done conducted in 22 countries, was terminated prematurely due to the significant adverse events and ineffectiveness on overall survival for rindopepimut (a vaccine targeting EGFRvIII) in newly diagnosed EGFRvIII+ glioblastoma patients who underwent maximal surgical resection and completed standard radiotherapy with concomitant temozolomide (12). However, another phase II, multicenter, prospective trial completed in American indicated that the toxicity of PEPvIII-KLH (another vaccine targeting EGFRvIII) was generally minimal in adults with newly diagnosed EGFRvIII+ glioblastoma patients who underwent maximal surgical resection and completed standard radiotherapy with concomitant temozolomide and the overall survival of patients with EGFRvIII specific antibody response was longer than that of patients without, and there was still a statistical difference after adjusting for factors such as age, Karnofsky performance status, and methylguanine methyltransferase methylation (13). ReACT, a double-blind, randomized, phase II study done in American, further confirmed the potential of rindopepimut for targeted immunotherapy among patients with recurrent EGFRvIII+ glioblastoma (14). Neurooncology Working Group of the German Cancer Society trial 16 (NOA-16), a non-controlled, open-label, single-arm, multicenter, first-in-humans phase I trial conducted in Germany, demonstrated that IDH1(R132H)-specific peptide vaccine (IDH1-vac) prolonged the pseudoprogression and survival time of patients with IDH1(R132H)+, non-1p/19q co-deleted, ATRX− World Health Organization (WHO) grade 3 and 4 gliomas and the vaccine-related adverse events were restricted to grade 1 (15). Moreover, the new antigen IDH1 (R132H) is immunogenic across multiple HLA alleles and effectively induced an IDH1-specific immune response (15). MultIceNTER Phase I Peptide VaCcine Trial for the Treatment of H3-Mutated Gliomas (INTERCEPT-H3), a non-controlled, open-label, single arm, multicenter phase I trial done in Germany, showed that H3K27M-specific long peptide vaccine (H3K27M-vac) induced neoepitope-specific CD4+ T cell-dominated colocalization immune responses with HLA class II-DR in patients with newly diagnosed H3K27M diffuse midline gliomas and these patients with immune responses showed radiographic improvement (16).

## Dendritic cell vaccine

Dendritic cell vaccine is a vaccine that utilizes the patient’s own dendritic cells to activate the immune system and enhance its ability to attack tumor cells. The main function of dendritic cells is to initiate and activate the recognition and response of initial T cells to protein antigens, present viral and tumor antigens. It is the only dedicated antigen presenting cell that can directly activate initial T cells. Several phase I clinical trials found that three biweekly intradermal administration of autologous tumor lysate-pulsed dendritic cell vaccine among malignant glioma patients elicited antigen-specific systemic and intracranial cytotoxic and memory T-cell infiltration (17–19). A later phase I/II clinical study done in Japan showed the safety and clinical response of autologous tumor lysate-pulsed dendritic cell therapy for patients with malignant glioma (20, 21). A phase 3 randomized, double-blinded, placebo-controlled clinical trial conducted in the American, Canada, Germany, and the United Kingdom showed that addition of autologous tumor lysate-pulsed dendritic cell vaccine (DCVax^®^-L) to standard therapy was feasible and safe in newly diagnosed or recurrent glioblastoma patients and extended progression free survival and overall survival (22, 23). A randomized, double-blind, multicenter phase II placebo-controlled study found autologous tumor lysate-pulsed dendritic cell vaccine (ICT-107) improved progression free survival and maintained quality of life in recurrent glioma patients (24). A single-arm phase 2 clinical trial completed in American indicated that the Aivita glioblastoma vaccine (AV-GBM-1) was well-tolerated and extended the median progression free survival with numerous treatment-emergent central nervous system adverse events (25). A single-center, randomized, open-label multi-arm phase II clinical trial conducted in American indicated that combining autologous tumor lysate-pulsed dendritic cell vaccine with poly-ICLC, a Toll-like receptor agonist, induced a polarized interferon activation in circulating monocytes and CD8+ T cells, which may represent an important blood biomarker for immunotherapy in patients with newly-diagnosed or recurrent WHO Grade III-IV malignant gliomas (26). There are different variants for dendritic cell vaccine. The cytomegalovirus-specific dendritic cell vaccine patients improved long-term progression free survival and overall survival in patients with newly diagnosed glioblastoma (27) and pre-conditioning the cytomegalovirus-specific dendritic cell vaccine site with a potent recall antigen significantly improves the lymph node homing and efficacy of vaccine (28), later sequential clinical trials utilizing cytomegalovirus-specific dendritic cell vaccine associated with extended overall survival for newly diagnosed glioblastoma (29). The other one is autologous dendritic cell vaccine pulsed with lysate derived from an allogeneic stem-like cell line, which was safe and well tolerated in newly diagnosed and recurrent glioblastoma (30).

## Tumor neoantigen vaccine

Neoantigen originates from tumor specific protein coding mutations and is not affected by central tolerance. It can generate strong immune responses and act as an unquestionable antigen to promote tumor rejection (31) (**Table 1**, **Supplementary Figure 1**). Single-cell T cell receptor analysis of phase I/Ib study involving personalized neoantigen-targeting vaccines for patients with newly diagnosed glioblastoma demonstrated neoantigen-specific T cells from the peripheral blood can migrate into intracranial glioblastoma (32). NeoVax, a personalized neoantigen-targeting vaccine, significantly increased neoantigen-specific effector T cells in newly diagnosed glioblastoma patients (33). These encouraging results contribute to the development of future clinical trials for glioma patients based on neoantigen vaccines.

**Table 1:** Some trials of tumor vaccines in glioma.

Trial	Participants	Sample size	Intervention	Study design	Results	Reference
	WT1+ malignant glioma	14	WT1 vaccine	phase I clinical study	Eleven of the 14 patients completed WT1 vaccination for 6 weeks, while 3 patients dropped out earlier due to disease progression. All patients showed grade I level of skin disorders at the injection sites. No grade III/IV toxicity or dose-limiting toxicity was observed for any dose of WT1 HLA class II peptide. Six of the 14 patients had stable disease at 6 weeks. Median OS and 1-year OS rates were 24.7 weeks and 36%, respectively.	(9)
	WT1/HLA-A*2402-positive recurrent glioblastoma multiforme	21	WT1 vaccine	phase II clinical trial	The protocol was well tolerated; only local erythema occurred at the WT1 vaccine injection site. The clinical responses were as follows: partial response in 2 patients, stable disease in 10 patients, and progressive disease in 9 patients. No patient had a complete response. The overall response rate (cases with complete or partial response) was 9.5%, and the disease control rate (cases with complete or partial response as well as those in which disease was stable) was 57.1%. The median progression-free survival (PFS) period was 20.0 weeks, and the 6-month (26-week) PFS rate was 33.3%.	(10)
ACT IV, NCT01480479	newly diagnosed EGFRvIII+ glioblastoma	405	Rindopepimut	randomized, double-blind, phase 3 study	There was no significant difference in overall survival for patients with MRD: median overall survival was 20·1 months (95% CI 18·5-22·1) in the rindopepimut group versus 20·0 months (18·1-21·9) in the control group (HR 1·01, 95% CI 0·79-1·30; p=0·93). The most common grade 3-4 adverse events for all 369 treated patients in the rindopepimut group versus 372 treated patients in the control group were: thrombocytopenia (32 [9%] vs 23 [6%]), fatigue (six [2%] vs 19 [5%]), brain oedema (eight [2%] vs 11 [3%]), seizure (nine [2%] vs eight [2%]), and headache (six [2%] vs ten [3%]). Serious adverse events included seizure (18 [5%] vs 22 [6%]) and brain oedema (seven [2%] vs 12 [3%]). 16 deaths in the study were caused by adverse events (nine [4%] in the rindopepimut group and seven [3%] in the control group), of which one-a pulmonary embolism in a 64-year-old male patient after 11 months of treatment-was assessed as potentially related to rindopepimut.	(12)
	newly diagnosed EGFRvIII+ glioblastoma	35	PEPvIII-KLH	phase II, multicenter, prospective trial	There were no symptomatic autoimmune reactions. The 6-month PFS rate after vaccination was 67% (95% CI, 40% to 83%) and after diagnosis was 94% (95% CI, 67% to 99%; n = 18). The median OS was 26.0 months (95% CI, 21.0 to 47.7 months). After adjustment for age and Karnofsky performance status, the OS of vaccinated patients was greater than that observed in a control group matched for eligibility criteria, prognostic factors, and temozolomide treatment (hazard ratio, 5.3; P = .0013; n = 17). The development of specific antibody (P = .025) or delayed-type hypersensitivity (P = .03) responses to EGFRvIII had a significant effect on OS. At recurrence, 82% (95% CI, 48% to 97%) of patients had lost EGFRvIII expression (P <.001).	(13)
ReACT, NCT01498328	relapsed, EGFRvIII+ glioblastoma	73	Rindopepimut	double-blind, randomized, phase II study	Rindopepimut toxicity included transient, low-grade local reactions. As primary endpoint, PFS6 was 28% (10/36) for rindopepimut compared with 16% (6/37) for control (P = 0.12, one-sided). Secondary and exploratory endpoints also favored the rindopepimut group including a statistically significant survival advantage [HR, 0.53; 95% confidence interval (CI), 0.32-0.88; two-sided log-rank P = 0.01], a higher ORR [30% (9/30) vs. 18% (6/34; P = 0.38)], median duration of response [7.8 months (95% CI, 3.5-22.2) vs. 5.6 (95% CI, 3.7-7.4)], and ability to discontinue steroids for ≥6 months [33% (6/18) vs. 0% (0/19)]. Eighty percent of rindopepimut-treated patients achieved robust anti-EGFRvIII titers (≥1:12,800), which were associated with prolonged survival (HR = 0.17; 95% CI, 0.07-0.45; P < 0.0001).	(14)
NOA-16, NCT02454634	IDH1(R132H)+, non-1p/19q co-deleted, ATRX− WHO grade 3 and 4 gliomas	33	IDH1-vac	non-controlled, open-label, single-arm, multicenter, first-in-humans phase I trial	The trial met its primary safety endpoint, with vaccine-related adverse events restricted to grade 1. Vaccine-induced immune responses were observed in 93.3% of patients across multiple MHC alleles. Three-year progression-free and death-free rates were 0.63 and 0.84, respectively. Patients with immune responses showed a two-year progression-free rate of 0.82. Two patients without an immune response showed tumour progression within two years of first diagnosis.	(15)
INTERCEPT-H3, NCT04808245	newly diagnosed K27M-mutant histone-3.1 (H3.1K27M) or histone-3.3 (H3.3K27M) diffuse midline gliomas	8	H3K27M-vac	active multicenter, phase I clinical trial	Repeat vaccinations with H3K27M-vac were safe and induced CD4+ T cell-dominated, mutation-specific immune responses in five of eight patients across multiple human leukocyte antigen types. Median progression-free survival after vaccination was 6.2 months and median overall survival was 12.8 months.	(16)
	recurrent malignant glioma	24	autologous tumor lysate-pulsed dendritic cell vaccine	phase I/II clinical study	The protocols were well tolerated with only local redness and swelling at the injection site in several cases. Clinical responses were as follows: 1 patient with partial response, 3 patients with minor response, 10 patients with stable disease, and 10 patients with progressive disease. The patients whose dendritic cells were matured with OK-432 had longer survival times than the dendritic cells from patients without OK-432 maturation. The patients with both intratumoral and intradermal administrations had a longer survival time than the patients with intradermal administration only. Increased ELISPOT and delayed-type hypersensitivity responses after vaccination could provide good laboratory markers to predict the clinical outcome of patients receiving dendritic cell vaccination. The overall survival of patients with grade 4 glioma was 480 days, which was significantly better than that in the control group.	(20)
NCT00045968	newly diagnosed or recurrent glioblastoma	331	DCVax^®^-L	phase 3 randomized, double-blinded, placebo-controlled clinical trial	Median OS (mOS) for the 232 patients with nGBM receiving DCVax-L was 19.3 (95% CI, 17.5-21.3) months from randomization (22.4 months from surgery) vs 16.5 (95% CI, 16.0-17.5) months from randomization in control patients (HR = 0.80; 98% CI, 0.00-0.94; P = .002). Survival at 48 months from randomization was 15.7% vs 9.9%, and at 60 months, it was 13.0% vs 5.7%. For 64 patients with rGBM receiving DCVax-L, mOS was 13.2 (95% CI, 9.7-16.8) months from relapse vs 7.8 (95% CI, 7.2-8.2) months among control patients (HR, 0.58; 98% CI, 0.00-0.76; P <.001). Survival at 24 and 30 months after recurrence was 20.7% vs 9.6% and 11.1% vs 5.1%, respectively. Survival was improved in patients with nGBM with methylated MGMT receiving DCVax-L compared with external control patients (HR, 0.74; 98% CI, 0.55-1.00; P = .03).	(22, 23)
NCT 01280552	newly diagnosed glioblastoma	124	ICT-107	randomized, double-blind, multicenter phase II placebo-controlled study	ICT-107 was well tolerated, with no difference in adverse events between the treatment and control groups. The primary endpoint, median overall survival (OS), favored ICT-107 by 2.0 months in the intent-to-treat (ITT) population but was not statistically significant. Progression-free survival (PFS) in the ITT population was significantly increased in the ICT-107 cohort by 2.2 months (P = 0.011). The frequency of HLA-A2 primary tumor antigen expression was higher than that for HLA-A1 patients, and HLA-A2 patients had higher immune response (via Elispot). HLA-A2 patients achieved a meaningful therapeutic benefit with ICT-107, in both the MGMT methylated and unmethylated prespecified subgroups, whereas only HLA-A1 methylated patients had an OS benefit.	(24)
NCT03400917	newly diagnosed glioblastoma	63	AV-GBM-1	single-arm phase 2 clinical trial	Success rates were 97% for both TIC production and monocyte collection. AV-GBM-1 was manufactured for 63/63 patients; 60 enrolled per ITT; 57 started AV-GBM-1. The most common AEs attributed to AV-GBM-1 were local injection site reactions (16%) and flu-like symptoms (10%). Treatment-emergent AEs included seizures (33%), headache (37%), and focal neurologic symptoms (28%). One patient discontinued AV-GBM-1 because of seizures. Median Progression-Free Survival (mPFS) and median Overall Survival (mOS) from ITT enrollment were 10.4 and 16.0 months, respectively. 2-year Overall Survival (OS) is 27%.	(25)
NCT01204684	newly diagnosed or recurrent WHO Grade III-IV malignant gliomas	23	dendritic cell vaccination	single-center, randomized, open-label multi-arm phase II clinical trial	The combination of ATL-DC vaccination and TLR agonist was safe and found to enhance systemic immune responses, as indicated by increased interferon gene expression and changes in immune cell activation.	(26)
ATTAC, NCT00639639	newly diagnosed glioblastoma	11	cytomegalovirus-specific dendritic cell vaccine	randomized phase I/II trial	Following DI-TMZ cycle 1 and three doses of pp65-DCs, pp65 cellular responses significantly increased. After DI-TMZ, both the proportion and proliferation of regulatory T cells (Tregs) increased and remained elevated with serial DI-TMZ cycles. Median PFS and OS were 25.3 months [95% confidence interval (CI), 11.0-∞] and 41.1 months (95% CI, 21.6-∞), exceeding survival using recursive partitioning analysis and matched historical controls. Four patients remained progression-free at 59 to 64 months from diagnosis. No known prognostic factors [age, Karnofsky performance status (KPS), IDH-1/2 mutation, and MGMT promoter methylation] predicted more favorable outcomes for the patients in this cohort.	(27)
NCT02287428	newly diagnosed MGMT-unmethylated glioblastoma	10	personalized neoantigen vaccines	phase I/Ib study	Patients who did not receive dexamethasone-a highly potent corticosteroid that is frequently prescribed to treat cerebral oedema in patients with glioblastoma-generated circulating polyfunctional neoantigen-specific CD4+ and CD8+ T cell responses that were enriched in a memory phenotype and showed an increase in the number of tumour-infiltrating T cells. Using single-cell T cell receptor analysis, we provide evidence that neoantigen-specific T cells from the peripheral blood can migrate into an intracranial glioblastoma tumour.	(32)
NCT03422094	newly diagnosed glioblastoma	4	NeoVax	single institution, open-label, multi-arm, pilot study	Immune reactivity to NeoVax neoantigens was assessed in peripheral blood mononuclear cells (PBMCs) pre- and post-NeoVax for subjects 1-3 using IFNg-ELISPOT assay. A statistically significant increase in IFNg producing T cells at the post-NeoVax time point for several neoantigens was observed. Furthermore, a post-NeoVax tumor biopsy was obtained from subject 3 and, upon evaluation, revealed evidence of infiltrating, clonally expanded T cells.	(33)

## Immune checkpoint inhibitors

Immune checkpoint inhibitors, including programmed cell death 1 (PD-1)/programmed cell death ligand 1 (PD-L1) and cytotoxic T lymphocyte-associated antigen 4 (CTLA-4), are important immunosuppressive targets for tumor escape (34)(**Table 2**, **Supplementary Figure 2**). The PD-1/PD-L1 axis promotes glioma tumor growth and invasion (35). CheckMate 143 confirmed the safety and tolerability of nivolumab in patients with newly diagnosed glioblastoma, median overall survival with nivolumab was 33.38 (16.2 to not estimable) and 16.49 (12.94-22.08) months in patients with methylated and unmethylated MGMT promoter, respectively (36). Later CheckMate 143 demonstrated median overall survival with nivolumab and bevacizumab was 9.8 (8.2-11.8) and 10.0 (9.0-11.8) months, respectively, in recurrent glioblastoma population, the objective response rate was 7.8% (4.1%-13.3%) and 23.1% (16.7%-30.5%) with nivolumab and with bevacizumab, respectively (37). Given the potential benefits of nivolumab for methylated patients, CheckMate 548 has emerged. However, CheckMate 548 did not achieve the primary endpoints with the median overall survival with nivolumab and placebo was 28.9 (24.4-31.6) and 32.1 (29.4-33.8) months, respectively, and the median progression free survival with nivolumab and placebo was 10.6 (8.9-11.8) and 10.3 (9.7-12.5) months, respectively (38). The above studies are all aimed at the basic treatment of simultaneous radiotherapy and temozolomide chemotherapy, while CheckMate 498 compared nivolumab with temozolomide chemotherapy on the basis of radiotherapy. The median overall survival with nivolumab and temozolomide chemotherapy was 13.4 (12.6-14.3) and 14.9 (13.3-16.1) months, respectively (hazard ratio =1.31 (1.09-1.58), P = 0.0037), and the median progression free survival with nivolumab and temozolomide chemotherapy was 6.0 (5.7-6.2) and 6.2 (5.9-6.7) months, respectively, in patients with newly diagnosed glioblastoma with unmethylated MGMT promoter (39). Changes in the immune status of gliomas may affect the patient’s responsiveness to anti-PD-1 immunotherapy (nivolumab or pembrolizumab) (40). Resectable glioblastoma tumor tissue pre- and post-nivolumab dosing resulted in higher immune cell infiltration, enhanced chemokine transcripts, and augmented T cell receptor clonal diversity, supporting a local immunomodulatory effect of treatment (41). Recurrent glioblastoma patients receiving neoadjuvant pembrolizumab had significantly extended overall survival, pembrolizumab was associated with upregulated T cell- and interferon-γ-related gene expression as well as downregulated cell-cycle-related gene expression, which enhancing both the intratumoral and systemic immune responses (42). The median survival of responders was longer than non-responders to neoadjuvant nivolumab or pembrolizumab in a retrospective analysis, MAPK pathway alterations were enriched in responders while PTEN mutations associated with immunosuppressive signature from CD44 + tumor cells were enriched in non-responders, and immune infiltration that reflect the tumor’s clonal evolution during treatment (43). Following 10 mg nivolumab intravenously administration, 27 recurrent glioblastoma patients underwent a maximal safe resection, followed by 10 mg ipilimumab or 5mg ipilimumab plus 10 mg nivolumab injection. The overall survival was better compared with historical cohorts (Belgian and GliAvAx trials) (44). CheckMate 908 investigated nivolumab and nivolumab plus ipilimumab in pediatric patients with high-grade central nervous system malignancies. The median overall survival with nivolumab and nivolumab plus ipilimumab was 1.7 (10.3-16.5) and 10.8 (9.1-15.8) months, respectively, in newly diagnosed diffuse intrinsic pontine glioma and the median progression free survival with nivolumab and nivolumab plus ipilimumab was 1.7 (1.4-2.7) and 1.3 (1.2-1.5) months, respectively, in high-grade glioma; 1.4 (1.2-1.4) and 2.8 (1.5-4.5) months, respectively, in medulloblastoma; 1.4 (1.4-2.6) and 4.6 (1.4-5.4) months in ependymoma; 1.2 (1.1-1.3) and 1.6 (1.3-3.5) months, respectively, in other recurrent/progressive central nervous system tumors (45). Bevacizumab could enhance the tolerability and efficacy of pembrolizumab in patients with recurrent high-grade gliomas receiving hypofractionated stereotactic irradiation. The median overall survival of bevacizumab-naïve and bevacizumab-resistant patients was 13.45 (9.46-18.46) and 9.3 (8.97-18.86) months, respectively, and the progression free survival was 7.92 (6.31-12.45) and 6.54 (5.95-18.86) months, respectively (46). Isatuximab plus atezolizumab had acceptable safety and tolerability and reduced CD38+ immune cells in the glioblastoma microenvironment (47).

**Table 2:** Some trials of immune checkpoint inhibitors in glioma.

Trial	Participants	Sample size	Intervention	Study design	Results	Reference
CheckMate 143, NCT02017717	newly diagnosed glioblastoma	136	nivolumab	phase 1 cohorts (1c+1d)	NIVO+RT ± TMZ was tolerable; grade 3/4 treatment-related adverse events occurred in 51.6% (NIVO+RT+TMZ) and 30.0% (NIVO+RT) of patients in part A and 46.4% (NIVO+RT+TMZ) and 28.6% (NIVO+RT) in part B. No new safety signals were detected. In part A, median OS (mOS) with NIVO+RT+TMZ was 33.38 months (95% CI, 16.2 to not estimable) in patients with methylated MGMT promoter. In patients with unmethylated MGMT promoter, mOS was 16.49 months (12.94-22.08) with NIVO+RT+TMZ and 14.41 months (12.55-17.31) with NIVO+RT. In part B, mOS was 14.75 months (10.01-18.6) with NIVO+RT+TMZ and 13.96 months (10.81-18.14) with NIVO+RT in patients with unmethylated MGMT promoter.	(36)
CheckMate 143, NCT02017717	recurrent glioblastoma	369	nivolumab	open-label, randomized, phase 3 clinical trial	The MGMT promoter was methylated in 23.4% (43/184; nivolumab) and 22.7% (42/185; bevacizumab), unmethylated in 32.1% (59/184; nivolumab) and 36.2% (67/185; bevacizumab), and not reported in remaining patients. At median follow-up of 9.5 months, median OS (mOS) was comparable between groups: nivolumab, 9.8 months (95% CI, 8.2-11.8); bevacizumab, 10.0 months (95% CI, 9.0-11.8); HR, 1.04 (95% CI, 0.83-1.30); P = .76. The 12-month OS was 42% in both groups. The objective response rate was higher with bevacizumab (23.1%; 95% CI, 16.7%-30.5%) vs nivolumab (7.8%; 95% CI, 4.1%-13.3%). Grade 3/4 treatment-related adverse events (TRAEs) were similar between groups (nivolumab, 33/182 [18.1%]; bevacizumab, 25/165 [15.2%]), with no unexpected neurological TRAEs or deaths due to TRAEs.	(37)
CheckMate 548, NCT02667587	newly diagnosed glioblastoma with methylated MGMT promoter	716	nivolumab	phase III, single-blind trial	As of December 22, 2020, median (m)PFS (blinded independent central review) was 10.6 months (95% CI, 8.9-11.8) with NIVO + RT + TMZ vs 10.3 months (95% CI, 9.7-12.5) with PBO + RT + TMZ (HR, 1.1; 95% CI, 0.9-1.3) and mOS was 28.9 months (95% CI, 24.4-31.6) vs 32.1 months (95% CI, 29.4-33.8), respectively (HR, 1.1; 95% CI, 0.9-1.3). In patients without baseline corticosteroids, mOS was 31.3 months (95% CI, 28.6-34.8) with NIVO + RT + TMZ vs 33.0 months (95% CI, 31.0-35.1) with PBO + RT + TMZ (HR, 1.1; 95% CI, 0.9-1.4). Grade 3/4 treatment-related adverse event rates were 52.4% vs 33.6%, respectively.	(38)
CheckMate 498, NCT02617589	newly diagnosed glioblastoma with unmethylated MGMT promoter	560	nivolumab	open-label, randomized, phase III study	Median OS (mOS) was 13.4 months (95% CI, 12.6 to 14.3) with NIVO + RT and 14.9 months (95% CI, 13.3 to 16.1) with TMZ + RT (hazard ratio [HR], 1.31; 95% CI, 1.09 to 1.58; P = .0037). Median progression-free survival was 6.0 months (95% CI, 5.7 to 6.2) with NIVO + RT and 6.2 months (95% CI, 5.9 to 6.7) with TMZ + RT (HR, 1.38; 95% CI, 1.15 to 1.65). Response rates were 7.8% (9/116) with NIVO + RT and 7.2% (8/111) with TMZ + RT; grade 3/4 treatment-related adverse event (TRAE) rates were 21.9% and 25.1%, and any-grade serious TRAE rates were 17.3% and 7.6%, respectively.	(39)
NCT02550249	resectable glioblastoma	30	nivolumab	single-arm phase II clinical trial	Neoadjuvant nivolumab resulted in enhanced expression of chemokine transcripts, higher immune cell infiltration and augmented TCR clonal diversity among tumor-infiltrating T lymphocytes, supporting a local immunomodulatory effect of treatment. Although no obvious clinical benefit was substantiated following salvage surgery, two of the three patients treated with nivolumab before and after primary surgery remain alive 33 and 28 months later.	(41)
	recurrent, surgically resectable glioblastoma	35	pembrolizumab	randomized, multi-institution clinical trial	Patients who were randomized to receive neoadjuvant pembrolizumab, with continued adjuvant therapy following surgery, had significantly extended overall survival compared to patients that were randomized to receive adjuvant, post-surgical programmed cell death protein 1 (PD-1) blockade alone. Neoadjuvant PD-1 blockade was associated with upregulation of T cell- and interferon-γ-related gene expression, but downregulation of cell-cycle-related gene expression within the tumor, which was not seen in patients that received adjuvant therapy alone. Focal induction of programmed death-ligand 1 in the tumor microenvironment, enhanced clonal expansion of T cells, decreased PD-1 expression on peripheral blood T cells and a decreasing monocytic population was observed more frequently in the neoadjuvant group than in patients treated only in the adjuvant setting.	(42)
	recurrent glioblastoma	66	pembrolizumab or nivolumab	retrospective analysis	Genomic and transcriptomic analysis revealed a significant enrichment of PTEN mutations associated with immunosuppressive expression signatures in non-responders, and an enrichment of MAPK pathway alterations (PTPN11, BRAF) in responders. Responsive tumors were also associated with branched patterns of evolution from the elimination of neoepitopes as well as with differences in T cell clonal diversity and tumor microenvironment profiles.	(43)
NCT03233152	recurrent glioblastoma	27	nivolumab and ipilimumab	single-center, open-label, phase I clinical trial	All patients underwent maximal safe resection and planned IC administrations and preoperative NIVO. Thirteen patients (cohort-1: n=3; cohort-2: n=10) received all five postoperative intravenous doses of NIVO. In cohort-2, 14 patients received a median of 3 (range 1-4) intravenous doses. Subacute postoperative neurological deterioration (n=2) was reversible on steroid treatment; no other central nervous system toxicity was observed. Immune-related adverse events were infrequent and mild. GB recurrence was diagnosed in 26 patients (median progression-free survival (PFS) is 11.7 weeks (range 2-152)); 21 patients have died due to progression. Median OS is 38 weeks (95% CI: 27 to 49) with a 6-month, 1-year, and 2-year OS-rate of, respectively, 74.1% (95% CI: 57 to 90), 40.7% (95% CI: 22 to 59), and 27% (95% CI: 9 to 44). OS compares favorable against a historical cohort (descriptive Log-Rank p>0.003). No significant difference was found with respect to PFS (descriptive Log-Rank test p>0.05). A higher tumor mRNA expression level of B7-H3 was associated with a significantly worse survival (multivariate Cox logistic regression, p>0.029).	(44)
CheckMate 908, NCT03130959	high-grade CNS malignancies	166	nivolumab and ipilimumab	open-label, sequential-arm, phase 1b/2 study	As of January 13, 2021, median OS (80% CI) was 11.7 (10.3-16.5) and 10.8 (9.1-15.8) months with NIVO and NIVO + IPI, respectively, in newly diagnosed DIPG. Median PFS (80% CI) with NIVO and NIVO + IPI was 1.7 (1.4-2.7) and 1.3 (1.2-1.5) months, respectively, in recurrent/progressive high-grade glioma; 1.4 (1.2-1.4) and 2.8 (1.5-4.5) months in relapsed/resistant medulloblastoma; and 1.4 (1.4-2.6) and 4.6 (1.4-5.4) months in relapsed/resistant ependymoma. In patients with other recurrent/progressive CNS tumors, median PFS (95% CI) was 1.2 (1.1-1.3) and 1.6 (1.3-3.5) months, respectively. Grade 3/4 treatment-related adverse-event rates were 14.1% (NIVO) and 27.2% (NIVO + IPI). NIVO and IPI first-dose trough concentrations were lower in youngest and lowest-weight patients. Baseline tumor programmed death ligand 1 expression was not associated with survival.	(45)
	recurrent high-grade gliomas	32	bevacizumab and pembrolizumab	phase I study	The most common treatment-related adverse events (TRAEs) were proteinuria (40.6%), fatigue (25%), increased alanine aminotransferase (25%), and hypertension (25%). TRAEs leading to discontinuation occurred in 1 patient who experienced a grade 3 elevation of aspartate aminotransferase. In the bevacizumab-naïve cohort, 20 patients (83%) had a complete response or partial response. The median overall survival (OS) and progression-free survival (PFS) were 13.45 months (95% CI: 9.46-18.46) and 7.92 months (95% CI: 6.31-12.45), respectively. In the bevacizumab-resistant cohort, PR was achieved in 5 patients (62%). Median OS was 9.3 months (95% CI: 8.97-18.86) with a median PFS of 6.54 months (95% CI: 5.95-18.86). The majority of patients (n = 20/26; 77%) had tumor-cell/tumor-microenvironment PD-L1 expression <1%.	(46)
NCT03637764	glioblastoma	33	isatuximab plus atezolizumab	phase I/II, open-label, multicenter study	In phase I, Isa + Atezo showed an acceptable safety profile, no dose-limiting toxicities were observed, and RP2D was confirmed. Most patients experienced ≥1 treatment-emergent adverse event (TEAE), with ≤48.5% being grade ≥3. The most frequent TEAE was infusion reactions. The study did not continue to stage 2 based on prespecified targets. Tumor-infiltrating CD38+ immune cells were reduced and almost cleared after treatment. Isa + Atezo did not significantly modulate Tregs or PD-L1 expression in the TME.	(47)

## CAR-T therapy

CAR-T can specifically recognize tumor cell surface antigens, independent of MHC activation, and produce stronger anti-tumor immune responses (48) (**Table 3**, **Supplementary Figure 3**). There are also many practices in gliomas. Among them, GD2, EGFR-vIII, HER2, and IL13Ra2 are the three most common targets and have been tested in early phase trial.

**Table 3:** Some trials of CAR-T therapy in glioma.

Trial	Participants	Sample size	Targets	Study design	Results	Reference
	recurrent or refractory advanced-stage neuroblastoma	11	GD2	Primary clinical trial	Here we show in individuals with neuroblastoma that EBV-specific CTLs expressing a chimeric GD2-specific receptor indeed survive longer than T cells activated by the CD3-specific antibody OKT3 and expressing the same chimeric receptor but lacking virus specificity. Infusion of these genetically modified cells seemed safe and was associated with tumor regression or necrosis in half of the subjects tested.	(49)
NCT04196413	diffuse midline gliomas	4	GD2	first-in-human phase I clinical trial	Toxicity was largely related to the location of the tumour and was reversible with intensive supportive care. On-target, off-tumour toxicity was not observed. Three of four patients exhibited clinical and radiographic improvement. Pro-inflammatory cytokine levels were increased in the plasma and cerebrospinal fluid. Transcriptomic analyses of 65,598 single cells from CAR T cell products and cerebrospinal fluid elucidate heterogeneity in response between participants and administration routes.	(50)
NCT03170141	glioblastoma	8	GD2	phase 1 trial	4SCAR-T cells expanded for 1-3 weeks and persisted at a low frequency in peripheral blood. Of the eight evaluable patients, four showed a partial response for 3 to 24 months, three had progressive disease for 6 to 23 months, and one had stable disease for 4 months after infusion. For the entire cohort, the median overall survival was 10 months from the infusion. GD2 antigen loss and infiltrated T cells were observed in the tumor resected after infusion.	(51)
NCT02209376	recurrent glioblastoma	10	EGFRvIII	phase 1 first-in-human study	We found that manufacturing and infusion of CAR-modified T cell (CART)-EGFRvIII cells are feasible and safe, without evidence of off-tumor toxicity or cytokine release syndrome.	(52)
NCT01454596	recurrent glioblastoma	33	EGFRvIII	dose-escalating phase I study	Eighteen patients were treated with final infusion products ranging from 6.3×10 to 2.6×10 anti-EGFRvIII CAR T cells. Median progression-free survival was 1.3 months (interquartile range: 1.1-1.9), with a single outlier of 12.5 months. Two patients experienced severe hypoxia, including one treatment-related mortality after cell administration at the highest dose level. All patients developed expected transient hematologic toxicities from preparative chemotherapy. Median overall survival was 6.9 months (interquartile range: 2.8-10). Two patients survived over 1 year, and a third patient was alive at 59 months. Persistence of CAR cells correlated with cell dose, but there were no objective responses.	(53)
NCT01109095	progressive glioblastoma	17	HER2	open-label phase 1 dose-escalation study	Infusions were well tolerated, with no dose-limiting toxic effects. HER2-CAR VSTs were detected in the peripheral blood for up to 12 months after the infusion by quantitative real-time polymerase chain reaction. Of 16 evaluable patients (9 adults and 7 children), 1 had a partial response for more than 9 months, 7 had stable disease for 8 weeks to 29 months, and 8 progressed after T-cell infusion. Three patients with stable disease are alive without any evidence of progression during 24 to 29 months of follow-up. For the entire study cohort, median overall survival was 11.1 months (95% CI, 4.1-27.2 months) from the first T-cell infusion and 24.5 months (95% CI, 17.2-34.6 months) from diagnosis.	(54)
NCT00730613	recurrent glioblastoma	3	IL13Rα2	single-institution first-in-human pilot study	We demonstrate the feasibility of manufacturing sufficient numbers of autologous CTL clones expressing an IL13(E13Y)-zetakine CAR for redirected HLA-independent IL13Rα2-specific effector function for a cohort of patients diagnosed with GBM. Intracranial delivery of the IL13-zetakine(+) CTL clones into the resection cavity of 3 patients with recurrent disease was well-tolerated, with manageable temporary brain inflammation. Following infusion of IL13-zetakine(+) CTLs, evidence for transient anti-glioma responses was observed in 2 of the patients. Analysis of tumor tissue from 1 patient before and after T-cell therapy suggested reduced overall IL13Rα2 expression within the tumor following treatment. MRI analysis of another patient indicated an increase in tumor necrotic volume at the site of IL13-zetakine(+) T-cell administration.	(55)
NCT02208362	recurrent malignant glioma	1	IL13Rα2	phase 1 study	Intracranial infusions of IL13Rα2-targeted CAR T cells were not associated with any toxic effects of grade 3 or higher. After CAR T-cell treatment, regression of all intracranial and spinal tumors was observed, along with corresponding increases in levels of cytokines and immune cells in the cerebrospinal fluid. This clinical response continued for 7.5 months after the initiation of CAR T-cell therapy.	(56)
NCT01082926	recurrent glioblastoma	6	IL13Rα2	phase 1, open-label, uncontrolled study	The GRm13Z40-2 product displayed dexamethasone-resistant effector activity without evidence for *in vitro* alloreactivity. Intracranial administration of GRm13Z40-2 in four doses of 108 cells over a two-week period with aldesleukin (9 infusions ranging from 2500-5000 IU) was well tolerated, with indications of transient tumor reduction and/or tumor necrosis at the site of T cell infusion in four of the six treated research subjects. Antibody reactivity against GRm13Z40-2 cells was detected in the serum of only one of the four tested subjects.	(57)

GD2-targeted CAR-T seemed safe in recurrent or refractory advanced-stage neuroblastoma and was associated with tumor regression or necrosis, thereby resulting in longer survival (49). GD2-targeted CAR-T was tolerable in H3K27M-mutated diffuse midline gliomas and improved the clinical and radiographic outcomes through the pro-inflammatory cytokine levels in the plasma and cerebrospinal fluid (50). Recent trial assessed the administration methods of GD2-targeted CAR-T and found that intravenous alone or intravenous combined with intracavitary administration of GD2-targeted CAR-T were safe and well tolerated in glioblastoma and GD2-targeted CAR-T mediated antigen loss and activated immune responses in the glioblastoma microenvironment (51). NCT02209376 was the first-in-human study of EGFRvIII-targeted CAR-T in recurrent glioblastoma. Intravenous infusion of EGFRvIII-targeted CAR-T was feasible and safe, increased inhibitory molecules expression and regulatory T cells infiltration (52). The dose-escalating phase I study indicated persistence of CAR-T correlated with EGFRvIII-targeted CAR-T dose, but there were no objective responses (53). Infusion of autologous HER2-targeted CAR-T was tolerated and was associated with clinical benefit for patients with progressive glioblastoma (54). NCT00730613 was the first-in-human pilot study to evaluate the safety and feasibility of IL13Rα2-targeted CAR-T in recurrent glioblastoma. Intracranial delivery of the IL13Rα2-targeted CAR-T was well-tolerated, induced transient anti-glioma responses, and increased tumor necrotic volume (55). A case report indicated intracranial infusion of IL13Rα2-targeted CAR-T was associated with no toxic effects of grade 3 or higher. After IL13Rα2-targeted CAR-T treatment, all intracranial and spinal tumors were regressed along with increased levels of cytokines and immune cells in the cerebrospinal fluid (56). Brown and his colleagues generated an off-the-shelf, steroid-resistant, IL13Rα2-targeted CAR-T and found it was safety and induced transient tumor reduction and/or tumor necrosis in patients with glioblastoma (57).

In addition to the targets reviewed above, CD7 (58) and EphA2 (59, 60) have conducted relevant research. Some research have also emerged. Local intracranial CAR-T elicits superior anti-tumor efficacy as compared to intravenous CAR-T, with intraventricular administration exhibiting possible benefits over intracranial administration in a multifocal disease model (61). Cyclophosphamide and fludarabine but not PD-1 inhibitor enhanced the expansion or persistence of GD2-targeted CAR-T (62). Trivalent CAR-T (HER2, IL13Rα2, and EphA2-targeted CAR-T) mediated immunoreaction forming polarized microtubule organizing centers, exhibited improved cytotoxicity and cytokine release, and overcame antigenic heterogeneity in glioblastoma thereby improving treatment outcomes (63). Moreover, HER2-targeted CAR-NK cells (64) and ErbB2-targeted CAR-NK cells (65) might be feasible and safe in recurrent glioblastoma. In a first-in-human trial published in 2024, three patients with recurrent glioblastoma received CARv3-TEAM-E T cell therapy (66). CARv3-TEAM-E T cells are chimeric antigen receptor (CAR) T cells that target both the epidermal growth factor receptor variant III (EGFRvIII), a tumor-specific antigen, and wild-type EGFR protein, attacking via secreted T cell-engaging antibody molecules (TEAM). The treatment did not cause any grade 3 or higher adverse events or dose-limiting toxicities. Radiological imaging revealed a rapid and significant reduction in tumor size, with responses occurring within days after a single intraventricular injection. However, two of the patients experienced only short-lived responses.

## Oncolytic virotherapy

Oncolytic viruses are a type of tumor-killing virus with replication ability. They not only proliferate infinitely within tumor cells, leading to their death, but also directly or indirectly activate the anti-tumor immune system, specifically killing tumor cells (67) (**Table 4**, **Supplementary Figure 4**). Due to its ability to recognize tumor cells specifically through corresponding receptors and its replication relying on tumor-specific promoters, oncolytic viruses do not affect normal brain tissue cells.

**Table 4:** Some trials of oncolytic virotherapy in glioma.

Trial	Participants	Adenovirus	Sample size	Oncolytic viruses	Study design	Results	Reference
	recurrent malignant gliomas	E1B	24	ONYX-015	phase I open-label, dose-escalation, multi-institutional trial	Adverse events were identified on physical exams and testing of hematologic, renal, and liver functions. Efficacy data were obtained from serial MRI scans. None of the 24 patients experienced serious adverse events related to ONYX-015. The maximum tolerated dose was not reached at 10(10) pfu. The median time to progression after treatment with ONYX-015 was 46 days (range 13 to 452 + days). The median survival time was 6.2 months (range 1.3 to 28.0 + months). One patient has not progressed and 1 patient showed regression of interval-increased enhancement. With more than 19 months of follow-up, 1/6 recipients at a dose of 10(9) and 2/6 at a dose of 10(10) pfu remain alive. In 2 patients who underwent a second resection 3 months after ONYX-015 injection, a lymphocytic and plasmacytoid cell infiltrate was observed. Injection of ONYX-015 into glioma cavities is well tolerated at doses up to 10(10) pfu.	(68)
NCT00805376	recurrent malignant gliomas	E1A	37	DNX-2401	phase I, dose-escalation, biologic-end-point clinical trial	In group A (n = 25), 20% of patients survived > 3 years from treatment, and three patients had a ≥ 95% reduction in the enhancing tumor (12%), with all three of these dramatic responses resulting in > 3 years of progression-free survival from the time of treatment. Analyses of post-treatment surgical specimens (group B, n = 12) showed that DNX-2401 replicates and spreads within the tumor, documenting direct virus-induced oncolysis in patients. In addition to radiographic signs of inflammation, histopathologic examination of immune markers in post-treatment specimens showed tumor infiltration by CD8+ and T-bet+ cells, and transmembrane immunoglobulin mucin-3 downregulation after treatment. Analyses of patient-derived cell lines for damage-associated molecular patterns revealed induction of immunogenic cell death in tumor cells after DNX-2401 administration.	(69)
NCT03178032	newly diagnosed diffuse intrinsic pontine glioma	E1A	12	DNX-2401	single-center, dose-escalation study	total of 12 patients, 3 to 18 years of age, with newly diagnosed DIPG received 1×1010 (the first 4 patients) or 5×1010 (the subsequent 8 patients) viral particles of DNX-2401, and 11 received subsequent radiotherapy. Adverse events among the patients included headache, nausea, vomiting, and fatigue. Hemiparesis and tetraparesis developed in 1 patient each. Over a median follow-up of 17.8 months (range, 5.9 to 33.5), a reduction in tumor size, as assessed on magnetic resonance imaging, was reported in 9 patients, a partial response in 3 patients, and stable disease in 8 patients. The median survival was 17.8 months. Two patients were alive at the time of preparation of the current report, 1 of whom was free of tumor progression at 38 months. Examination of a tumor sample obtained during autopsy from 1 patient and peripheral-blood studies revealed alteration of the tumor microenvironment and T-cell repertoire.	(73)
NCT02798406	recurrent glioblastoma	E1A	49	DNX-2401	two-part, phase 1/2, multicenter, open-label clinical trial	The primary endpoints were overall safety and objective response rate. The primary safety endpoint was met, whereas the primary efficacy endpoint was not met. There were no dose-limiting toxicities, and full dose combined treatment was well tolerated. The objective response rate was 10.4% (90% confidence interval (CI) 4.2-20.7%), which was not statistically greater than the prespecified control rate of 5%. The secondary endpoint of overall survival at 12 months was 52.7% (95% CI 40.1-69.2%), which was statistically greater than the prespecified control rate of 20%. Median overall survival was 12.5 months (10.7-13.5 months). Objective responses led to longer survival (hazard ratio 0.20, 95% CI 0.05-0.87). A total of 56.2% (95% CI 41.1-70.5%) of patients had a clinical benefit defined as stable disease or better. Three patients completed treatment with durable responses and remain alive at 45, 48 and 60 months.	(74)
NCT01491893	recurrent malignant glioma	CD155	61	PVSRIPO	phase 1 clinical trial with dose expansion	Dose level -1 (5.0×107 TCID50) was identified as the phase 2 dose. One dose-limiting toxic effect was observed; a patient in whom dose level 5 (1010 TCID50) was administered had a grade 4 intracranial hemorrhage immediately after the catheter was removed. To mitigate locoregional inflammation of the infused tumor with prolonged glucocorticoid use, dose level 5 was deescalated to reach the phase 2 dose. In the dose-expansion phase, 19% of the patients had a PVSRIPO-related adverse event of grade 3 or higher. Overall survival among the patients who received PVSRIPO reached a plateau of 21% (95% confidence interval, 11 to 33) at 24 months that was sustained at 36 months.	(75)
NCT03072134	newly diagnosed malignant gliomas	neural stem cells	12	NSC-CRAd-S-pk7	first-in-human, open-label, phase 1, dose-escalation trial	Histopathological evaluation identified 11 (92%) of 12 patients with glioblastoma and one (8%) of 12 patients with anaplastic astrocytoma. The median follow-up was 18 months (IQR 14-22). One patient receiving 1·50 × 108 NSCs loading 1·875 × 1011 viral particles developed viral meningitis (grade 3) due to the inadvertent injection of NSC-CRAd-S-pk7 into the lateral ventricle. Otherwise, treatment was safe as no formal dose-limiting toxicity was reached, so 1·50 × 108 NSCs loading 1·875 × 1011 viral particles was recommended as a phase 2 trial dose. There were no treatment-related deaths. The median progression-free survival was 9·1 months (95% CI 8·5-not reached) and median overall survival was 18·4 months (15·7-not reached).	(76)

A phase I dose-escalating clinical study showed that 24 patients with recurrent malignant gliomas received intracerebral injections of ONYX-015, an E1B-attenuated adenovirus. None of the patients had any adverse events related to ONYX-015 injection, confirming the relative safety of ONYX-015 in the treatment of malignant gliomas (68). DNX-2401, an E1A-attenuated adenovirus, resulted in more than 3 years of progression free survival in 12% of patients with recurrent malignant gliomas that were probably due to direct oncolytic effects of DNX-2401 followed by elicitation of an immune-mediated response (69, 70). DNX-2401 promoted a pro-inflammatory microenvironment and M1 characteristics of tumoral macrophages in glioblastoma (71), a later phase I clinical trial also indicated that DNX-2401 locally delivered by convection-enhanced delivery in tumor and surrounding brain induced local inflammatory reaction in patients with recurrent glioblastoma (72). DNX-2401 is also used in conjunction with other treatment methods. Intertumoral infusion of DNX-2401 followed by radiotherapy in pediatric patients with newly diagnosed diffuse intrinsic pontine glioma resulted in changes in T-cell activity and a reduction in or stabilization of tumor size in some patients but was associated with adverse events (73). The first-in-human investigation of combined DNX-2401 with pembrolizumab for recurrent glioblastoma confirmed the safety with notable survival benefits in select patients and response to treatment informed by the balance between immune infiltration and expression of immune checkpoint inhibitors (74). In addition, intratumoral infusion of PVSRIPO, recombinant nonpathogenic polio-rhinovirus chimera, in patients with recurrent malignant glioma confirmed the absence of neurovirulent potential and improved survival (75). A first-in-human, open-label, phase 1, dose-escalation trial indicated neural stem cell delivery of an oncolytic adenovirus (NSC-CRAd-S-pk7) in newly diagnosed malignant gliomas was feasible and safe (76). Ling AL, Solomon IH, Landivar AM, et al. report a phase I clinical trial linking oncolytic virus-mediated immune activation to survival in glioblastoma patients. In this study, 41 patients with recurrent glioblastoma were treated with CAN-3110, an oncolytic herpes virus designed for preferential tumor replication. No dose-limiting toxicities were observed. Improved survival was notably associated with HSV1 seropositivity, which also correlated with enhanced T cell response and immune activation signatures in the tumor microenvironment. These findings provide evidence supporting the therapeutic potential of oncolytic viruses in immunosuppressive tumors like glioblastoma (77).

## Discussion

Glioma is one of the most concerning fields in neurological tumors, and its treatment is a clinical challenge. Although the standard post-surgical treatment for newly diagnosed glioblastoma, involving concurrent radiotherapy and the alkylating chemotherapeutic agent temozolomide (TMZ) followed by adjuvant TMZ, has been established for over a decade, glioblastoma inevitably recurs and develops resistance to further chemotherapy. One of the earliest mechanisms of resistance to TMZ is the upregulation of DNA methyltransferase (MGMT), which removes methyl adducts from DNA, enabling mismatch repair and allowing tumor DNA replication to continue (78). Immunotherapy is a research hotspot, and many immunotherapy studies are targeting glioma. Here, we reviewed the current concepts of immunotherapy in glioma (**Tables 1**–**4**), the current clinical research results may be encouraging, but there is still much room for improvement.

Tumor vaccines activate T cells in patients by introducing tumor antigens, thereby inducing an immune response to kill tumor cells. Compared with traditional radiotherapy and chemotherapy, tumor vaccines have become highly promising immunotherapy due to their convenient operation process, strong specificity, good safety, and ability to establish long-term immune memory. The core of tumor vaccine development lies in accurately screening suitable tumor antigens and determining effective antigen delivery methods. To overcome these challenges, researchers have proposed various strategies. Firstly, the combination of tumor vaccines and immune checkpoint inhibitors is used to enhance immune response, thereby enhancing treatment efficacy. NEO-PV-01, a personalized neoantigen vaccine, in combination with pembrolizumab, supports the safety and immunogenicity in patients with advanced non-squamous non-small cell lung cancer (79). Secondly, the personalized vaccine strategy based on neoantigens improves the targeting and efficacy of tumor vaccines by accurately identifying tumor-specific antigens. The future research focus will be on combining clinical efficacy and artificial intelligence technology to improve the accuracy of predicting neoantigens, thereby optimizing the therapeutic effect and application scope of vaccines. Finally, utilizing systems biology methods could optimize vaccine design and improve vaccine delivery systems. By utilizing advanced biomaterials such as nanoparticles to optimize drug delivery systems could improve the vaccine efficacy and reduce side effects (80). KK2DP7, a simple dendrimer polypeptide nanoparticle, enhances antitumor immunity of a neoantigen-based vaccine (81). CRISPR-Cas9 technology enhances the immune response by precisely modifying the genetic information of immune cells or tumor cells. The engineered therapeutic tumor cells, repurposed from interferon-β sensitive to resistant using CRISPR-Cas9 by knocking out the interferon-β-specific receptor, eliminated established glioblastoma tumors in mice (82).

The research progress on immune checkpoint inhibitors is rapid, especially monoclonal antibodies targeting PD-1/PD-L1 and CTLA-4, which have been approved by the FDA for first-line treatment of melanoma and/or lung cancer. At the same time, research is also underway, including targeting LAG3, TIM3, and other checkpoint inhibitors. The combination immunotherapy method of PD-1 blockade was successfully used for advanced glioma. However, intratumoral heterogeneity (83, 84), low PD-L1 expression (85), low mutation burden (86), and chemotherapy-induced mutation properties (87, 88) in diffuse midline glioma might explain why no survival benefits have been observed with immune checkpoint inhibitors monotherapy (16, 89). Therefore, the FDA has not yet approved malignant glioma as a treatment indication for immune checkpoint inhibitors. In the future, further research is needed on neuroimmunology and the possibility of combining immune checkpoint inhibitors with other therapies to treat glioma.

Due to the heterogeneity of glioma and the immune suppression of CAR-T by the tumor microenvironment, increasing the number of CAR-T coverage antigens and combination therapy is key to improving the efficacy of CAR-T. Combination therapy with Lp2-targeted CAR-T and oncolytic virus G47Δ further inhibited the glioblastoma growth and improved survival (90). Although CAR-T treatment for glioma is still in its early stages, the emergence and application of advanced biotechnology will accelerate the search for new strategies for glioma. The method of genome-scale screening using CRISPR-Cas9 can significantly shorten the time for discovering key genes that can be interfered with to improve the therapeutic effect of CAR-T, a three-dimensional model of glioblastoma organoids that summarizes the cellular heterogeneity, structure, and function of primary tissues can be used for better preclinical studies of CAR-T therapy, and single-cell sequencing technology can accurately reveal the intratumoral heterogeneity of glioma cells and other cellular components of tumor microenvironment to provide rich information for monitoring immune response and predict therapeutic efficacy during the treatment process.

The progress of basic research and the iteration of technology have made oncolytic viruses more specific, effective, and safe in the treatment of glioma. More and more oncolytic viruses are entering phase I, II, and even III clinical trials. However, there are still many challenges in the treatment of glioma with oncolytic viruses. Firstly, in terms of safety, although existing clinical trial results have not reported significant safety events, there is still a risk of off-target treatment. In addition, although genetically modified viruses enhance their ability to recognize tumor cells, elderly people and immunocompromised patients may still be infected with the virus and cause serious consequences. Secondly, in terms of drug delivery routes, most oncolytic viruses are currently administered locally through direct intratumoral injection in the treatment of gliomas, reducing the risk of virus replication in non-target cells (91). However, there are problems such as bleeding, infection, and difficulty in drug delivery in deep lesions, and continuous improvement of targeted guidance techniques and the technical standards for intratumoral drug delivery should be emphasized. Compared with direct intratumoral injection, intravenous injection is undoubtedly more convenient and safer (92). However, due to the dilution of systemic blood, rapid neutralization of antibodies, and isolation of non-target organs and the blood-brain barrier, the application of intravenous medication is greatly limited. Increasing the dose of oncolytic viruses used for intravenous injection, improving oncolytic virus vectors, and using new ultrasound to open the blood-brain barrier (93) may improve the effectiveness of intravenous administration. Finally, how to improve the therapeutic effect of oncolytic viruses is also a difficult problem. Attention should be paid to the application of combining oncolytic virotherapy with other treatments such as chemotherapy, radiotherapy, and immune checkpoint inhibitors in the treatment of glioma to increase anti-tumor synergistic effects. Because there are currently no biomarkers available to predict the dose of relevant oncolytic viruses and their potential for *in vivo* replication (94), in addition to central imaging review, molecular pathology, and immune monitoring, in-depth research on virus replication and clinical anti-tumor response should be conducted in preclinical models and clinical trials.

The challenge of immunotherapy for glioma is still limited by objective factors. From an anatomical perspective, it is difficult to obtain central nervous system tumor section specimens, and due to the presence of the blood-brain barrier, some drugs are difficult to accurately reach the tumor periphery. To address anatomical difficulties, it is possible to simultaneously obtain tumor pathological tissue specimens on the basis of maximizing tumor tissue resection, and analyze individual tumor phenotype characteristics using multiple immunohistochemistry, proteomics, spatial transcriptomics techniques, etc. It is also possible to collect tumor-derived circulating DNA from cerebrospinal fluid before surgical resection, supplemented by tumor genome analysis, to obtain immunotherapy target information for a certain individual. Local administration (intratumoral delivery) may be feasible for addressing the challenges of the blood-brain barrier. Meanwhile, with the development of nanotechnology, it can cross the blood-brain barrier and target tumor sites, bringing new ways for immunotherapy of glioma. From the perspective of peritumoral characteristics, the immunosuppressive microenvironment is dynamic, which poses challenges to micro level research. Enhanced monitoring of individual patients may help capture immune dynamics, and non-invasive methods may be urgently needed due to increased frequency. With the widespread application of second-generation sequencing technology, the current research direction is mainly to search for new tumor mutations and antigens; in addition, research on immune related pathways and immunosuppressive mechanisms is also a new direction in tumor immunotherapy. Gliomas also exhibit a tendency to recur, further limiting the effectiveness of immunotherapy. It is possible to enhance the function of memory lymphocytes, search for specific receptors on the surface of memory lymphocytes, promote the function of activating receptors, and block the function of inhibitory receptors.

In addition, clinical research design also needs improvement. In clinical trials, the number of glioma patients is relatively small compared to more common solid tumors, which requires new therapies, including immunotherapy, to be tested in small, underpowered, and non-randomized designs. Nearly half of the published clinical studies are single-arm studies with a sample size of no more than 50 patients. These limitations make it difficult to demonstrate the efficacy of new therapies in glioma. Low randomization rate, insufficient use of the controls, and overestimation of benefits/effects, especially in early trials, may limit the widespread applicability of the results. The suboptimal design may be driven by accrual challenges, emphasizing the need for more collaborative efforts and creating incentives in these areas to enable larger-scale experiments, ideally using synchronous controls, but in appropriate cases, inequal randomization can be considered. The use of historical data can lead to bias in patient selection. Therefore, it is recommended to conduct randomized controlled clinical studies. Another area that needs improvement is the outcome indicators. Response rate is not an ideal endpoint for immunotherapy. Similarly, progression free survival is often used, but progression free survival also has the same issue in terms of response rate dependent on MRI evaluation, which may be inaccurate as pseudoprogression and pseudoresponse are common in glioblastoma. The Response Assessment in Neuro-Oncology criteria may provide some assistance. The most powerful clinical endpoint is overall survival. However, overall survival need longer follow-up time. Finally, the regulatory and technical authorities for trials should strengthen their supervision of the design and implementation of trials, and if necessary, terminate trials promptly to ensure that trials are designed and conducted in a safe, effective, and feasible manner for glioma patients.

Overall, despite the uneven quality and mixed results of available clinical trials, we still see the dawn of immunotherapy for glioma. Molecular biomarkers are of great significance in providing assistance and evaluating prognosis (95). With the advancement of cutting-edge technologies (artificial intelligence, CRISPR-Cas9, and single-cell sequencing), not only new evaluation methods can be provided for the immunotherapy of glioma, but more importantly, it is conducive to the rapid development of new immunotherapy strategies. The field of clinical trials of immunotherapy may contribute to individualized and personalized treatment and ultimately be applied in clinical practice, achieving the ultimate goal of improving the prognosis of patients with glioma (96).

## References

[1] Weller M Wick W Aldape K Brada M Berger M Pfister SM. Glioma. Nat Rev Dis Primers. (2015) 1:15017. doi: 10.1038/nrdp.2015.17 27188790

[2] Forjaz G Barnholtz-Sloan JS Kruchko C Siegel R Negoita S Ostrom QT. An updated histology recode for the analysis of primary Malignant and nonmalignant brain and other central nervous system tumors in the Surveillance, Epidemiology, and End Results Program. Neurooncol Adv. (2021) 3:vdaa175. doi: 10.1093/noajnl/vdaa175 33506208 PMC7813198

[3] Ostrom QT Gittleman H Liao P Rouse C Chen Y Dowling J. CBTRUS statistical report: primary brain and central nervous system tumors diagnosed in the United States in 2007-2011. Neuro Oncol. (2014) 16 Suppl 4:iv1–63. doi: 10.1093/neuonc/nou223 PMC4193675 25304271

[4] Weller M Cloughesy T Perry JR Wick W. Standards of care for treatment of recurrent glioblastoma–are we there yet? Neuro-oncology. (2013) 15:4–27. doi: 10.1093/neuonc/nos273 23136223 PMC3534423

[5] Huang Y Wang Y Huang Z. A specific peptide vaccine against IDH1(R132H) glioma. Neurosci Bull. (2022) 38:223–5. doi: 10.1007/s12264-021-00791-9 PMC8821749 34739683

[6] Old LJ Clarke DA Benacerraf B. Effect of Bacillus Calmette-Guerin infection on transplanted tumours in the mouse. Nature. (1959) 184:291–2. doi: 10.1038/184291a0 14428599

[7] Lin MJ Svensson-Arvelund J Lubitz GS Marabelle A Melero I Brown BD. Cancer vaccines: the next immunotherapy frontier. Nat Cancer. (2022) 3:911–26. doi: 10.1038/s43018-022-00418-6 35999309

[8] Saxena M van der Burg SH Melief CJM Bhardwaj N. Therapeutic cancer vaccines. Nat Rev Cancer. (2021) 21:360–78. doi: 10.1038/s41568-021-00346-0 33907315

[9] Tsuboi A Hashimoto N Fujiki F Morimoto S Kagawa N Nakajima H. A phase I clinical study of a cocktail vaccine of Wilms' tumor 1 (WT1) HLA class I and II peptides for recurrent Malignant glioma. Cancer Immunol Immunother. (2019) 68:331–40. doi: 10.1007/s00262-018-2274-1 PMC6394509 30430205

[10] Izumoto S Tsuboi A Oka Y Suzuki T Hashiba T Kagawa N. Phase II clinical trial of Wilms tumor 1 peptide vaccination for patients with recurrent glioblastoma multiforme. J Neurosurg. (2008) 108:963–71. doi: 10.3171/JNS/2008/108/5/0963 18447714

[11] Yokota C Kagawa N Takano K Chiba Y Kinoshita M Kijima N. Maintenance of WT1 expression in tumor cells is associated with a good prognosis in Malignant glioma patients treated with WT1 peptide vaccine immunotherapy. Cancer Immunology Immunotherapy. (2022) 71:189–201. doi: 10.1007/s00262-021-02954-z 34089373 PMC10991314

[12] Weller M Butowski N Tran DD Recht LD Lim M Hirte H. Rindopepimut with temozolomide for patients with newly diagnosed, EGFRvIII-expressing glioblastoma (ACT IV): a randomised, double-blind, international phase 3 trial. Lancet Oncol. (2017) 18:1373–85. doi: 10.1016/S1470-2045(17)30517-X 28844499

[13] Sampson JH Heimberger AB Archer GE Aldape KD Friedman AH Friedman HS. Immunologic escape after prolonged progression-free survival with epidermal growth factor receptor variant III peptide vaccination in patients with newly diagnosed glioblastoma. J Clin Oncol. (2010) 28:4722–9. doi: 10.1200/JCO.2010.28.6963 PMC3020702 20921459

[14] Reardon DA Desjardins A Vredenburgh JJ O'Rourke DM Tran DD Fink KL. Rindopepimut with bevacizumab for patients with relapsed EGFRvIII-expressing glioblastoma (ReACT): results of a double-blind randomized phase II trial. Clin Cancer Res. (2020) 26:1586–94. doi: 10.1158/1078-0432.CCR-18-1140 32034072

[15] Platten M Bunse L Wick A Bunse T Cornet LL Harting I. A vaccine targeting mutant IDH1 in newly diagnosed glioma. Nature. (2021) 592:463–8. doi: 10.1038/s41586-021-03363-z PMC8046668 33762734

[16] Grassl N Poschke I Lindner K Bunse L Mildenberger I Boschert T. A H3K27M-targeted vaccine in adults with diffuse midline glioma. Nat Med. (2023) 29:2586–92. doi: 10.1038/s41591-023-02555-6 PMC10579055 37735561

[17] Liau LM Prins RM Kiertscher SM Odesa SK Kremen TJ Giovannone AJ. Dendritic cell vaccination in glioblastoma patients induces systemic and intracranial T-cell responses modulated by the local central nervous system tumor microenvironment. Clin Cancer Res. (2005) 11:5515–25. doi: 10.1158/1078-0432.CCR-05-0464 16061868

[18] Yu JS Wheeler CJ Zeltzer PM Ying H Finger DN Lee PK. Vaccination of Malignant glioma patients with peptide-pulsed dendritic cells elicits systemic cytotoxicity and intracranial T-cell infiltration. Cancer Res. (2001) 61:842–7. 11221866

[19] Yu JS Liu G Ying H Yong WH Black KL Wheeler CJ. Vaccination with tumor lysate-pulsed dendritic cells elicits antigen-specific, cytotoxic T-cells in patients with Malignant glioma. Cancer Res. (2004) 64:4973–9. doi: 10.1158/0008-5472.CAN-03-3505 15256471

[20] Yamanaka R Homma J Yajima N Tsuchiya N Sano M Kobayashi T. Clinical evaluation of dendritic cell vaccination for patients with recurrent glioma: results of a clinical phase I/II trial. Clin Cancer Res. (2005) 11:4160–7. doi: 10.1158/1078-0432.CCR-05-0120 15930352

[21] Yamanaka R Abe T Yajima N Tsuchiya N Homma J Kobayashi T. Vaccination of recurrent glioma patients with tumour lysate-pulsed dendritic cells elicits immune responses: results of a clinical phase I/II trial. Br J Cancer. (2003) 89:1172–9. doi: 10.1038/sj.bjc.6601268 PMC2394324 14520441

[22] Liau LM Ashkan K Tran DD Campian JL Trusheim JE Cobbs CS. First results on survival from a large Phase 3 clinical trial of an autologous dendritic cell vaccine in newly diagnosed glioblastoma. J Transl Med. (2018) 16:142. doi: 10.1186/s12967-018-1507-6 29843811 PMC5975654

[23] Liau LM Ashkan K Brem S Campian JL Trusheim JE Iwamoto FM. Association of autologous tumor lysate-loaded dendritic cell vaccination with extension of survival among patients with newly diagnosed and recurrent glioblastoma: A phase 3 prospective externally controlled cohort trial. JAMA Oncol. (2023) 9:112–21. doi: 10.1001/jamaoncol.2022.5370 PMC9673026 36394838

[24] Wen PY Reardon DA Armstrong TS Phuphanich S Aiken RD Landolfi JC. A randomized double-blind placebo-controlled phase II trial of dendritic cell vaccine ICT-107 in newly diagnosed patients with glioblastoma. Clin Cancer Res. (2019) 25:5799–807. doi: 10.1158/1078-0432.CCR-19-0261 PMC8132111 31320597

[25] Bota DA Taylor TH Piccioni DE Duma CM LaRocca RV Kesari S. Phase 2 study of AV-GBM-1 (a tumor-initiating cell targeted dendritic cell vaccine) in newly diagnosed Glioblastoma patients: safety and efficacy assessment. J Exp Clin Cancer Res. (2022) 41:344. doi: 10.1186/s13046-022-02552-6 36517865 PMC9749349

[26] Everson RG Hugo W Sun L Antonios J Lee A Ding L. TLR agonists polarize interferon responses in conjunction with dendritic cell vaccination in Malignant glioma: a randomized phase II Trial. Nat Commun. (2024) 15:3882. doi: 10.1038/s41467-024-48073-y 38719809 PMC11078958

[27] Batich KA Reap EA Archer GE Sanchez-Perez L Nair SK Schmittling RJ. Long-term survival in glioblastoma with cytomegalovirus pp65-targeted vaccination. Clin Cancer Res. (2017) 23:1898–909. doi: 10.1158/1078-0432.CCR-16-2057 PMC5559300 28411277

[28] Mitchell DA Batich KA Gunn MD Huang MN Sanchez-Perez L Nair SK. Tetanus toxoid and CCL3 improve dendritic cell vaccines in mice and glioblastoma patients. Nature. (2015) 519:366–9. doi: 10.1038/nature14320 PMC4510871 25762141

[29] Batich KA Mitchell DA Healy P Herndon JE II Sampson JH. Once, twice, three times a finding: reproducibility of dendritic cell vaccine trials targeting cytomegalovirus in glioblastoma. Clin Cancer Res. (2020) 26:5297–303. doi: 10.1158/1078-0432.CCR-20-1082 PMC9832384 32719000

[30] Hu JL Omofoye OA Rudnick JD Kim S Tighiouart M Phuphanich S. A phase I study of autologous dendritic cell vaccine pulsed with allogeneic stem-like cell line lysate in patients with newly diagnosed or recurrent glioblastoma. Clin Cancer Res. (2022) 28:689–96. doi: 10.1158/1078-0432.CCR-21-2867 34862245

[31] Peng M Mo Y Wang Y Wu P Zhang Y Xiong F. Neoantigen vaccine: an emerging tumor immunotherapy. Mol Cancer. (2019) 18:128. doi: 10.1186/s12943-019-1055-6 31443694 PMC6708248

[32] Keskin DB Anandappa AJ Sun J Tirosh I Mathewson ND Li S. Neoantigen vaccine generates intratumoral T cell responses in phase Ib glioblastoma trial. Nature. (2019) 565:234–9. doi: 10.1038/s41586-018-0792-9 PMC6546179 30568305

[33] Johanns TM Garfinkle EAR Miller KE Livingstone AJ Roberts KF Venkata LPR. Integrating multisector molecular characterization into personalized peptide vaccine design for patients with newly diagnosed glioblastoma. Clin Cancer Res. (2024) 30(13):2729–42. doi: 10.1158/1078-0432.27034444.v1 PMC11215407 38639919

[34] Zhou JG Liang B Liu JG Jin SH He SS Frey 2B. Identification of 15 lncRNAs signature for predicting survival benefit of advanced melanoma patients treated with anti-PD-1 monotherapy. Cells. (2021) 10(5):977. doi: 10.3390/cells10050977 33922038 PMC8143567

[35] Wang XP Guo W Chen YF Hong C Ji J Zhang XY. PD-1/PD-L1 axis is involved in the interaction between microglial polarization and glioma. Int Immunopharmacol. (2024) 133:112074. doi: 10.1016/j.intimp.2024.112074 38615383

[36] Omuro A Reardon DA Sampson JH Baehring J Sahebjam S Cloughesy TF. Nivolumab plus radiotherapy with or without temozolomide in newly diagnosed glioblastoma: Results from exploratory phase I cohorts of CheckMate 143. Neurooncol Adv. (2022) 4:vdac025. doi: 10.1093/noajnl/vdac025 35402913 PMC8989388

[37] Reardon DA Brandes AA Omuro A Mulholland P Lim M Wick A. Effect of nivolumab vs bevacizumab in patients with recurrent glioblastoma: the checkMate 143 phase 3 randomized clinical trial. JAMA Oncol. (2020) 6:1003–10. doi: 10.1001/jamaoncol.2020.1024 PMC7243167 32437507

[38] Lim M Weller M Idbaih A Steinbach J Finocchiaro G Raval RR. Phase III trial of chemoradiotherapy with temozolomide plus nivolumab or placebo for newly diagnosed glioblastoma with methylated MGMT promoter. Neuro-Oncology. (2022) 24:1935–49. doi: 10.1093/neuonc/noac116 PMC9629431 35511454

[39] Omuro A Brandes AA Carpentier AF Idbaih A Reardon DA Cloughesy T. Radiotherapy combined with nivolumab or temozolomide for newly diagnosed glioblastoma with unmethylated MGMT promoter: An international randomized phase III trial. Neuro-Oncology. (2022) 25:123–34. doi: 10.1093/neuonc/noac099 PMC9825306 35419607

[40] Ito H Nakashima H Chiocca EA. Molecular responses to immune checkpoint blockade in glioblastoma. Nat Med. (2019) 25:359–61. doi: 10.1038/s41591-019-0385-7 PMC6742426 30842671

[41] Schalper KA Rodriguez-Ruiz ME Diez-Valle R López-Janeiro A Porciuncula A Idoate MA. Neoadjuvant nivolumab modifies the tumor immune microenvironment in resectable glioblastoma. Nat Med. (2019) 25:470–6. doi: 10.1038/s41591-018-0339-5 30742120

[42] Cloughesy TF Mochizuki AY Orpilla JR Hugo W Lee AH Davidson TB. Neoadjuvant anti-PD-1 immunotherapy promotes a survival benefit with intratumoral and systemic immune responses in recurrent glioblastoma. Nat Med. (2019) 25:477–86. doi: 10.1038/s41591-018-0337-7 PMC6408961 30742122

[43] Zhao J Chen AX Gartrell RD Silverman AM Aparicio L Chu T. Immune and genomic correlates of response to anti-PD-1 immunotherapy in glioblastoma. Nat Med. (2019) 25:462–9. doi: 10.1038/s41591-019-0349-y PMC6810613 30742119

[44] Duerinck J Schwarze JK Awada G Tijtgat J Vaeyens F Bertels C. Intracerebral administration of CTLA-4 and PD-1 immune checkpoint blocking monoclonal antibodies in patients with recurrent glioblastoma: a phase I clinical trial. J Immunother Cancer. (2021) 9:e002296. doi: 10.1136/jitc-2020-002296 34168003 PMC8231061

[45] Dunkel IJ Doz F Foreman NK Hargrave D Lassaletta A André N. Nivolumab with or without ipilimumab in pediatric patients with high-grade CNS Malignancies: Safety, efficacy, biomarker, and pharmacokinetics-CheckMate 908. Neuro Oncol. (2023) 25:1530–45. doi: 10.1093/neuonc/noad031 PMC10398811 36808285

[46] Sahebjam S Forsyth PA Tran ND Arrington JA Macaulay R Etame AB. Hypofractionated stereotactic re-irradiation with pembrolizumab and bevacizumab in patients with recurrent high-grade gliomas: results from a phase I study. Neuro Oncol. (2021) 23:677–86. doi: 10.1093/neuonc/noaa260 PMC8041351 33173935

[47] Simonelli M Garralda E Eskens F Gil-Martin M Yen CJ Obermannova R. Isatuximab plus atezolizumab in patients with advanced solid tumors: results from a phase I/II, open-label, multicenter study. ESMO Open. (2022) 7:100562. doi: 10.1016/j.esmoop.2022.100562 35987165 PMC9588873

[48] Brown MP Ebert LM Gargett T. Clinical chimeric antigen receptor-T cell therapy: a new and promising treatment modality for glioblastoma. Clin Transl Immunol. (2019) 8:e1050. doi: 10.1002/cti2.2019.8.issue-5 PMC6526894 31139410

[49] Pule MA Savoldo B Myers GD Rossig C Russell HV Dotti G. Virus-specific T cells engineered to coexpress tumor-specific receptors: persistence and antitumor activity in individuals with neuroblastoma. Nat Med. (2008) 14:1264–70. doi: 10.1038/nm.1882 PMC2749734 18978797

[50] Majzner RG Ramakrishna S Yeom KW Patel S Chinnasamy H Schultz LM. GD2-CAR T cell therapy for H3K27M-mutated diffuse midline gliomas. Nature. (2022) 603:934–41. doi: 10.1038/s41586-022-04489-4 PMC8967714 35130560

[51] Liu Z Zhou J Yang X Liu Y Zou C Lv W. Safety and antitumor activity of GD2-Specific 4SCAR-T cells in patients with glioblastoma. Mol Cancer. (2023) 22:3. doi: 10.1186/s12943-022-01711-9 36617554 PMC9827625

[52] O’Rourke DM Nasrallah MP Desai A Melenhorst JJ Mansfield K Morrissette JJD. A single dose of peripherally infused EGFRvIII-directed CAR T cells mediates antigen loss and induces adaptive resistance in patients with recurrent glioblastoma. Sci Trans Med. (2017) 9:eaaa0984. doi: 10.1126/scitranslmed.aaa0984 PMC5762203 28724573

[53] Goff SL Morgan RA Yang JC Sherry RM Robbins PF Restifo NP. Pilot trial of adoptive transfer of chimeric antigen receptor-transduced T cells targeting EGFRvIII in patients with glioblastoma. J Immunother. (2019) 42:126–35. doi: 10.1097/CJI.0000000000000260 PMC6691897 30882547

[54] Ahmed N Brawley V Hegde M Bielamowicz K Kalra M Landi D. HER2-specific chimeric antigen receptor–modified virus-specific T cells for progressive glioblastoma: A phase 1 dose-escalation trial. JAMA Oncol. (2017) 3:1094–101. doi: 10.1001/jamaoncol.2017.0184 PMC5747970 28426845

[55] Brown CE Badie B Barish ME Weng L Ostberg JR Chang WC. Bioactivity and safety of IL13Rα2-redirected chimeric antigen receptor CD8+ T cells in patients with recurrent glioblastoma. Clin Cancer Res. (2015) 21:4062–72. doi: 10.1158/1078-0432.CCR-15-0428 PMC4632968 26059190

[56] Brown Christine E Alizadeh D Starr R Weng L Wagner JR Naranjo A. Regression of glioblastoma after chimeric antigen receptor T-cell therapy. New Engl J Med. (2016) 375:2561–9. doi: 10.1056/NEJMoa1610497 PMC5390684 28029927

[57] Brown CE Rodriguez A Palmer J Ostberg JR Naranjo A Wagner JR. Off-the-shelf, steroid-resistant, IL13Rα2-specific CAR T cells for treatment of glioblastoma. Neuro-Oncology. (2022) 24:1318–30. doi: 10.1093/neuonc/noac024 PMC9340633 35100373

[58] Jin L Ge H Long Y Yang C Chang YE Mu L. CD70, a novel target of CAR T-cell therapy for gliomas. Neuro Oncol. (2018) 20:55–65. doi: 10.1093/neuonc/nox116 28651374 PMC5761579

[59] Chow KK Naik S Kakarla S Brawley VS Shaffer DR Yi Z. T cells redirected to EphA2 for the immunotherapy of glioblastoma. Mol Ther. (2013) 21:629–37. doi: 10.1038/mt.2012.210 PMC3589173 23070117

[60] Lin Q Ba T Ho J Chen D Cheng Y Wang L. First-in-human trial of ephA2-redirected CAR T-cells in patients with recurrent glioblastoma: A preliminary report of three cases at the starting dose. Front Oncol. (2021) 11:694941. doi: 10.3389/fonc.2021.694941 34235085 PMC8256846

[61] Brown CE Aguilar B Starr R Yang X Chang WC Weng L. Optimization of IL13Rα2-targeted chimeric antigen receptor T cells for improved anti-tumor efficacy against glioblastoma. Mol Ther. (2018) 26:31–44. doi: 10.1016/j.ymthe.2017.10.002 29103912 PMC5763077

[62] Heczey A Louis CU Savoldo B Dakhova O Durett A Grilley B. CAR T cells administered in combination with lymphodepletion and PD-1 inhibition to patients with neuroblastoma. Mol Ther. (2017) 25:2214–24. doi: 10.1016/j.ymthe.2017.05.012 PMC5589058 28602436

[63] Bielamowicz K Fousek K Byrd TT Samaha H Mukherjee M Aware N. Trivalent CAR T cells overcome interpatient antigenic variability in glioblastoma. Neuro Oncol. (2018) 20:506–18. doi: 10.1093/neuonc/nox182 PMC5909636 29016929

[64] Burger MC Forster MT Romanski A Straßheimer F Macas J Zeiner PS. Intracranial injection of natural killer cells engineered with a HER2-targeted chimeric antigen receptor in patients with recurrent glioblastoma. Neuro Oncol. (2023) 25:2058–71. doi: 10.1093/neuonc/noad087 PMC10628939 37148198

[65] Zhang C Burger MC Jennewein L Genßler S Schönfeld K Zeiner P. ErbB2/HER2-specific NK cells for targeted therapy of glioblastoma. J Natl Cancer Inst. (2016) 108(5). doi: 10.1093/jnci/djv375 26640245

[66] Choi BD Gerstner ER Frigault MJ Leick MB Mount CW Balaj L. Intraventricular CARv3-TEAM-E T cells in recurrent glioblastoma. New Engl J Med. (2024) 390:1290–8. doi: 10.1056/NEJMoa2314390 PMC11162836 38477966

[67] Wang JL Scheitler KM Wenger NM Elder JB. Viral therapies for glioblastoma and high-grade gliomas in adults: a systematic review. Neurosurg Focus. (2021) 50:E2. doi: 10.3171/2020.11.FOCUS20854 33524943

[68] Chiocca EA Abbed KM Tatter S Louis DN Hochberg FH Barker F. A phase I open-label, dose-escalation, multi-institutional trial of injection with an E1B-attenuated adenovirus, ONYX-015, into the peritumoral region of recurrent Malignant gliomas, in the adjuvant setting. Mol Ther. (2004) 10:958–66. doi: 10.1016/j.ymthe.2004.07.021 15509513

[69] Lang FF Conrad C Gomez-Manzano C Yung WKA Sawaya R Weinberg JS. Phase I study of DNX-2401 (Delta-24-RGD) oncolytic adenovirus: replication and immunotherapeutic effects in recurrent Malignant glioma. J Clin Oncol. (2018) 36:1419–27. doi: 10.1200/JCO.2017.75.8219 PMC6075856 29432077

[70] Martínez-Vélez N Garcia-Moure M Marigil M González-Huarriz M Puigdelloses M Pérez-Larraya JG. The oncolytic virus Delta-24-RGD elicits an antitumor effect in pediatric glioma and DIPG mouse models. Nat Commun. (2019) 10:2235. doi: 10.1038/s41467-019-10043-0 31138805 PMC6538754

[71] van den Bossche WBL Kleijn A Teunissen CE Voerman JSA Teodosio C Noske DP. Oncolytic virotherapy in glioblastoma patients induces a tumor macrophage phenotypic shift leading to an altered glioblastoma microenvironment. Neuro Oncol. (2018) 20:1494–504. doi: 10.1093/neuonc/noy082 PMC6176807 29796615

[72] van Putten EHP Kleijn A van Beusechem VW Noske D Lamers CHJ Goede ALD. Convection enhanced delivery of the oncolytic adenovirus delta24-RGD in patients with recurrent GBM: A phase I clinical trial including correlative studies. Clin Cancer Res. (2022) 28:1572–85. doi: 10.1158/1078-0432.CCR-21-3324 PMC9365362 35176144

[73] Gállego Pérez-Larraya J Garcia-Moure M Labiano S Patiño-García A Dobbs J Gonzalez-Huarriz M. Oncolytic DNX-2401 virus for pediatric diffuse intrinsic pontine glioma. N Engl J Med. (2022) 386:2471–81. doi: 10.1056/NEJMoa2202028 35767439

[74] Nassiri F Patil V Yefet LS Singh O Liu J Dang RMA. Oncolytic DNX-2401 virotherapy plus pembrolizumab in recurrent glioblastoma: a phase 1/2 trial. Nat Med. (2023) 29:1370–8. doi: 10.1038/s41591-023-02347-y PMC10287560 37188783

[75] Desjardins A Gromeier M Herndon JE 2nd Beaubier N Bolognesi DP Friedman AH. Recurrent glioblastoma treated with recombinant poliovirus. N Engl J Med. (2018) 379:150–61. doi: 10.1056/NEJMoa1716435 PMC6065102 29943666

[76] Fares J Ahmed AU Ulasov IV Sonabend AM Miska J Lee-Chang C. Neural stem cell delivery of an oncolytic adenovirus in newly diagnosed Malignant glioma: a first-in-human, phase 1, dose-escalation trial. Lancet Oncol. (2021) 22:1103–14. doi: 10.1016/S1470-2045(21)00245-X PMC8328944 34214495

[77] Ling AL Solomon IH Landivar AM Nakashima H Woods JK Santos A. Clinical trial links oncolytic immunoactivation to survival in glioblastoma. Nature. (2023) 623:157–66. doi: 10.1038/s41586-023-06623-2 PMC10620094 37853118

[78] Ou A Yung WKA Majd N. Molecular mechanisms of treatment resistance in glioblastoma. Int J Mol Sci. (2020) 22(1):351. doi: 10.3390/ijms22010351 33396284 PMC7794986

[79] Awad MM Govindan R Balogh KN Spigel DR Garon EB Bushway ME. Personalized neoantigen vaccine NEO-PV-01 with chemotherapy and anti-PD-1 as first-line treatment for non-squamous non-small cell lung cancer. Cancer Cell. (2022) 40:1010–26.e11. doi: 10.1016/j.ccell.2022.08.003 36027916

[80] Liu Z Shi M Ren Y Xu H Weng S Ning W. Recent advances and applications of CRISPR-Cas9 in cancer immunotherapy. Mol Cancer. (2023) 22:35. doi: 10.1186/s12943-023-01738-6 36797756 PMC9933290

[81] Zhang R Tang L Wang Y Tian Y Wu S Zhou B. A dendrimer peptide (KK2DP7) delivery system with dual functions of lymph node targeting and immune adjuvants as a general strategy for cancer immunotherapy. Adv Sci (Weinh). (2023) 10:e2300116. doi: 10.1002/advs.202300116 36950751 PMC10214225

[82] Chen KS Reinshagen C Van Schaik TA Rossignoli F Borges P Mendonca NC. Bifunctional cancer cell-based vaccine concomitantly drives direct tumor killing and antitumor immunity. Sci Transl Med. (2023) 15:eabo4778. doi: 10.1126/scitranslmed.abo4778 36599004 PMC10068810

[83] Johnson KC Anderson KJ Courtois ET Gujar AD Barthel FP Varn FS. Single-cell multimodal glioma analyses identify epigenetic regulators of cellular plasticity and environmental stress response. Nat Genet. (2021) 53:1456–68. doi: 10.1038/s41588-021-00926-8 PMC8570135 34594038

[84] Vinci M Burford A Molinari V Popov S Clarke M Taylor KR. Functional diversity and cooperativity between subclonal populations of pediatric glioblastoma and diffuse intrinsic pontine glioma cells. Nat Med. (2018) 24:1204–15. doi: 10.1038/s41591-018-0086-7 PMC6086334 29967352

[85] Jha P Manjunath N Singh J Mani K Garg A Kaur K. Analysis of PD-L1 expression and T cell infiltration in different molecular subgroups of diffuse midline gliomas. Neuropathology. (2019) 39:413–24. doi: 10.1111/neup.12594 31625205

[86] Alexandrov LB Nik-Zainal S Wedge DC Aparicio SAJR Behjati S Biankin AV. Signatures of mutational processes in human cancer. Nature. (2013) 500:415–21. doi: 10.1038/nature12477 PMC3776390 23945592

[87] Touat M Li YY Boynton AN Spurr LF Iorgulescu JB Bohrson CL. Mechanisms and therapeutic implications of hypermutation in gliomas. Nature. (2020) 580:517–23. doi: 10.1038/s41586-020-2209-9 PMC8235024 32322066

[88] McGranahan N Furness AJ Rosenthal R Ramskov S Lyngaa R Saini SK. Clonal neoantigens elicit T cell immunoreactivity and sensitivity to immune checkpoint blockade. Science. (2016) 351:1463–9. doi: 10.1126/science.aaf1490 PMC4984254 26940869

[89] Cacciotti C Choi J Alexandrescu S Zimmerman MA Cooney TM Chordas C. Immune checkpoint inhibition for pediatric patients with recurrent/refractory CNS tumors: a single institution experience. J Neurooncol. (2020) 149:113–22. doi: 10.1007/s11060-020-03578-6 32627129

[90] Chalise L Kato A Ohno M Maeda ZS Yamamichi A Kuramitsu S. Efficacy of cancer-specific anti-podoplanin CAR-T cells and oncolytic herpes virus G47Δ combination therapy against glioblastoma. Mol Ther Oncolytics. (2022) 26:265–74. doi: 10.1016/j.omto.2022.07.006 PMC9364057 35991754

[91] Harrington K Freeman DJ Kelly B Harper J Soria JC. Optimizing oncolytic virotherapy in cancer treatment. Nat Rev Drug Discovery. (2019) 18:689–706. doi: 10.1038/s41573-019-0029-0 31292532

[92] LaRocca CJ Warner SG. Oncolytic viruses and checkpoint inhibitors: combination therapy in clinical trials. Clin Transl Med. (2018) 7:35. doi: 10.1186/s40169-018-0214-5 30426287 PMC6234197

[93] Sonabend AM Gould A Amidei C Ward R Schmidt KA Zhang DY. Repeated blood-brain barrier opening with an implantable ultrasound device for delivery of albumin-bound paclitaxel in patients with recurrent glioblastoma: a phase 1 trial. Lancet Oncol. (2023) 24:509–22. doi: 10.1016/S1470-2045(23)00112-2 PMC10256454 37142373

[94] Kaufman HL Kohlhapp FJ Zloza A. Oncolytic viruses: a new class of immunotherapy drugs. Nat Rev Drug Discovery. (2015) 14:642–62. doi: 10.1038/nrd4663 PMC7097180 26323545

[95] Louis DN Perry A Wesseling P Brat DJ Cree IA Figarella-Branger D. The 2021 WHO classification of tumors of the central nervous system: a summary. Neuro-oncology. (2021) 23:1231–51. doi: 10.1093/neuonc/noab106 PMC8328013 34185076

[96] Zhang P Zhang Y Ji N. Challenges in the treatment of glioblastoma by chimeric antigen receptor T-cell immunotherapy and possible solutions. Front Immunol. (2022) 13:927132. doi: 10.3389/fimmu.2022.927132 35874698 PMC9300859

