# Supplementary figures and images for: Clinical immunotherapy in glioma: current concepts, challenges, and future perspectives
Source: Front Immunol. 2024 Nov 1;15:1476436. doi: 10.3389/fimmu.2024.1476436 (PMC11564147; doi:10.3389/fimmu.2024.1476436)

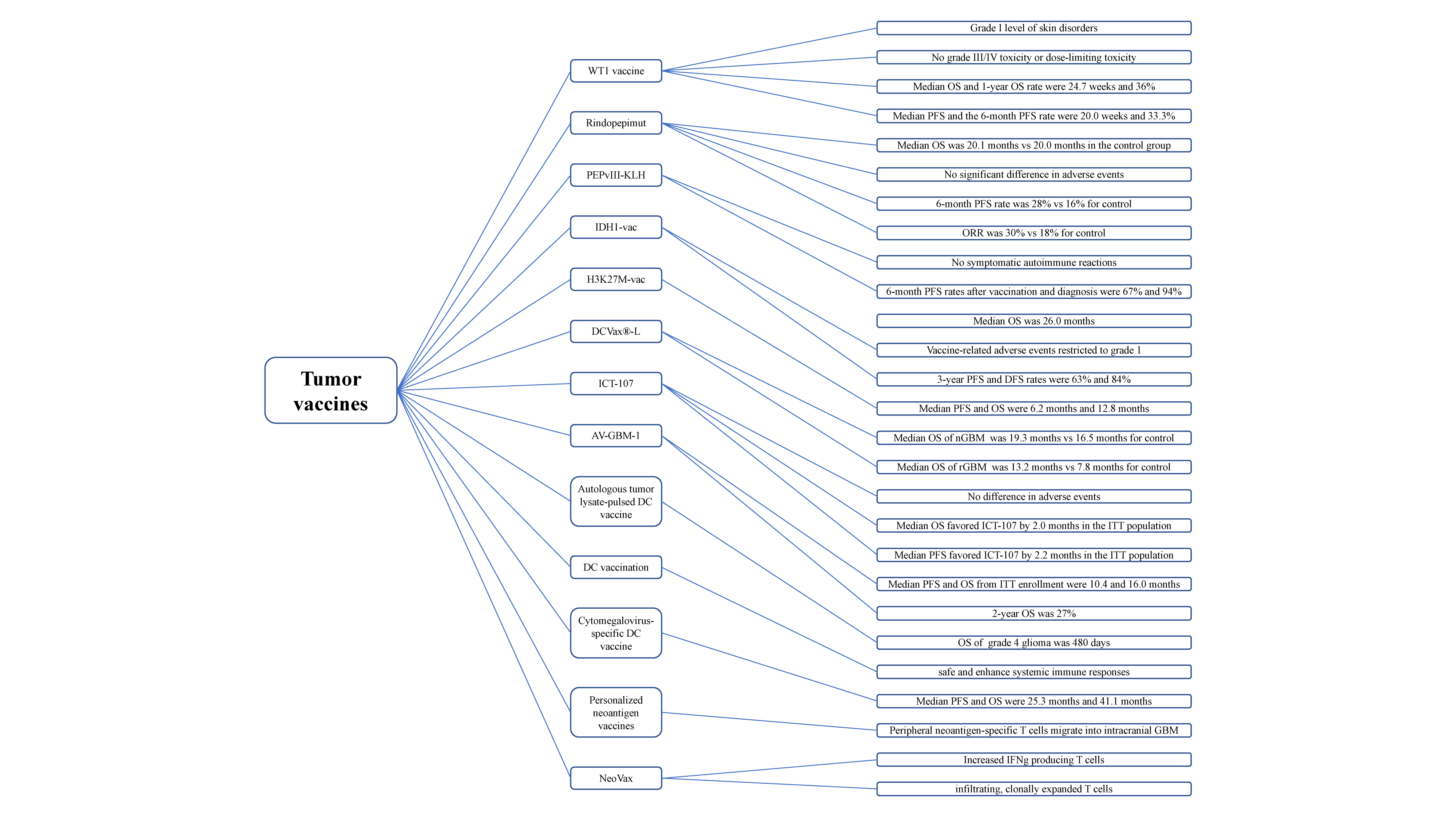

Supplement: Supplementary file 1 [file Image1.tif]

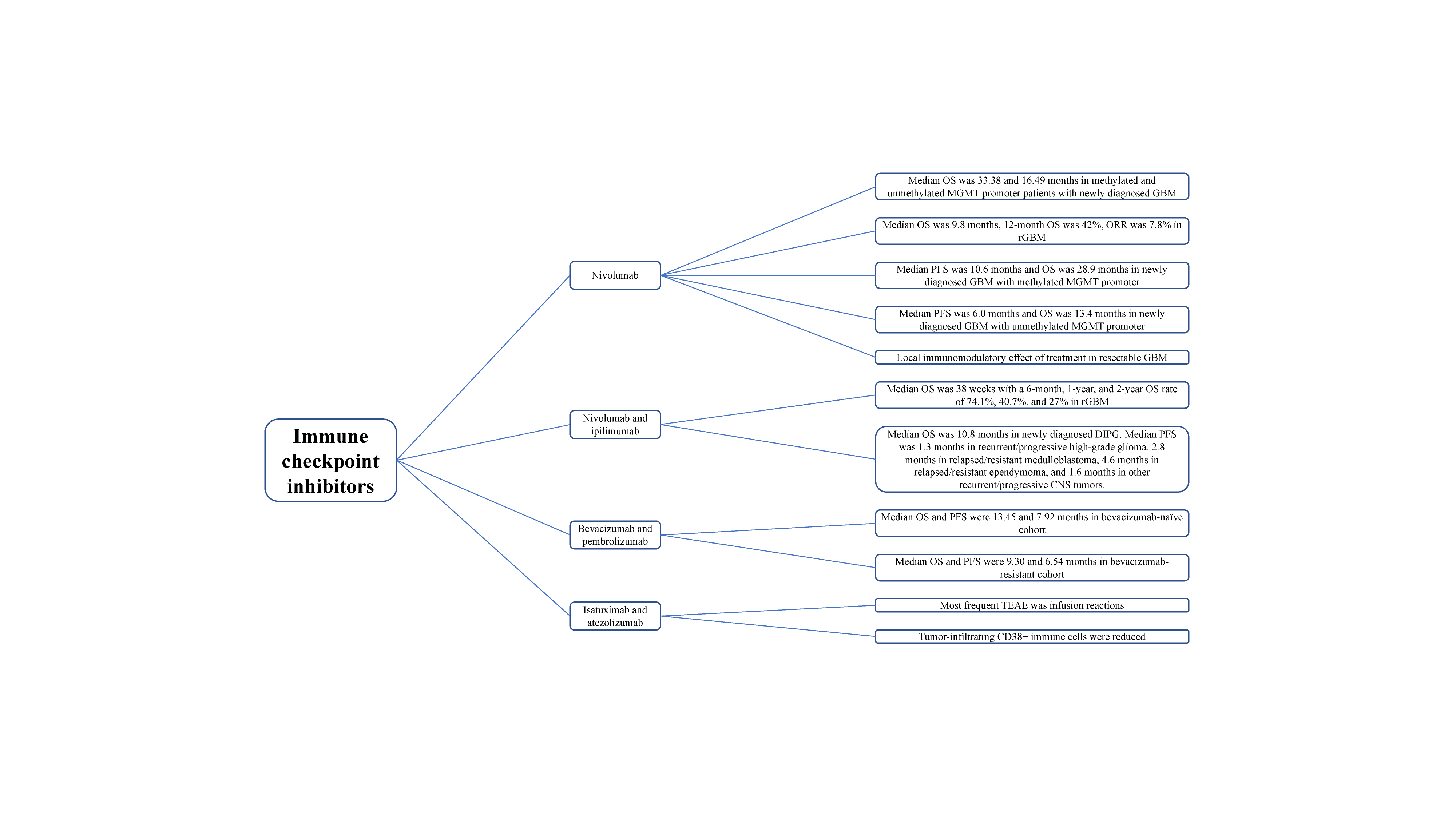

Supplement: Supplementary file 2 [file Image2.tif]

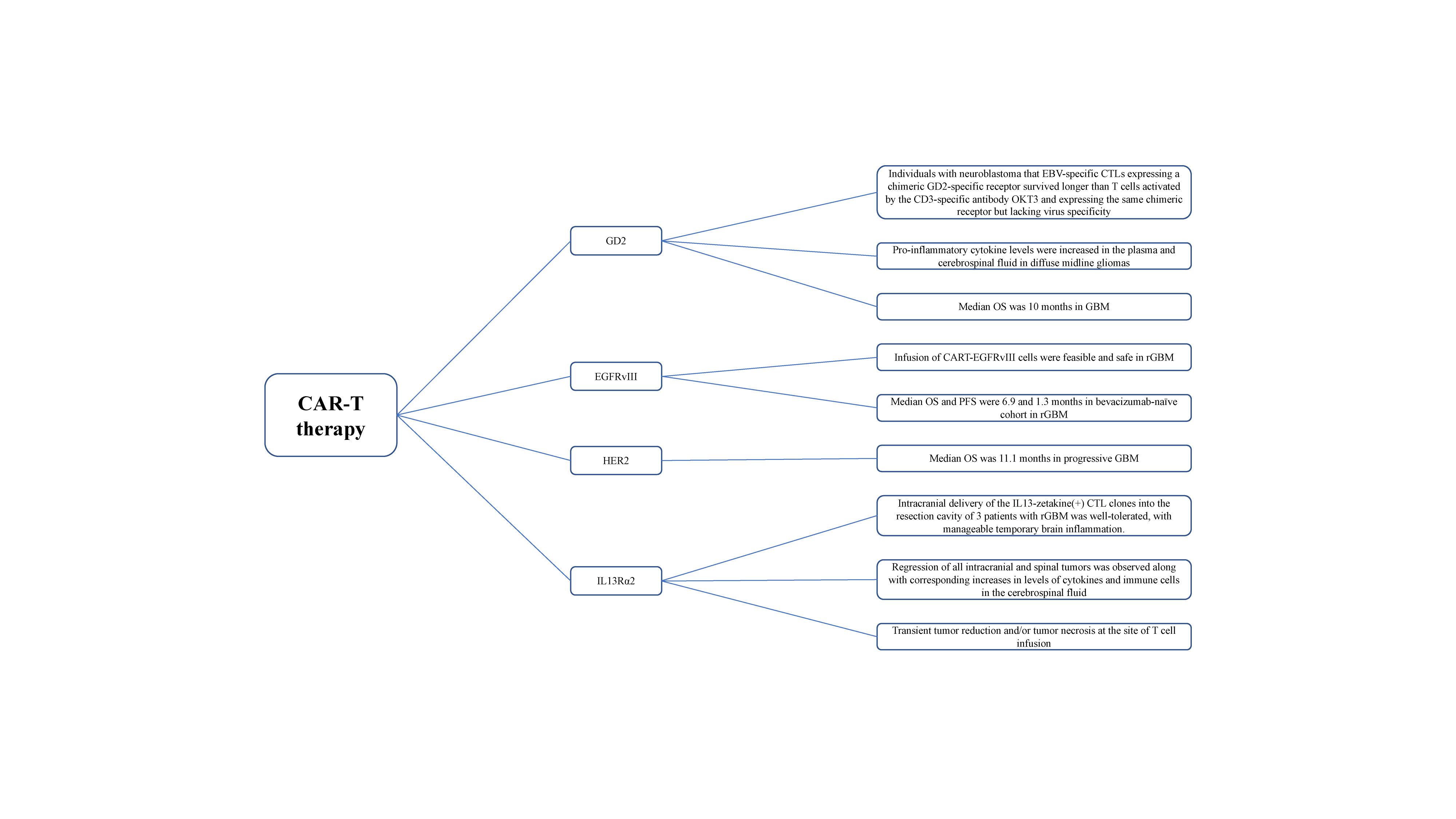

Supplement: Supplementary file 3 [file Image3.tif]

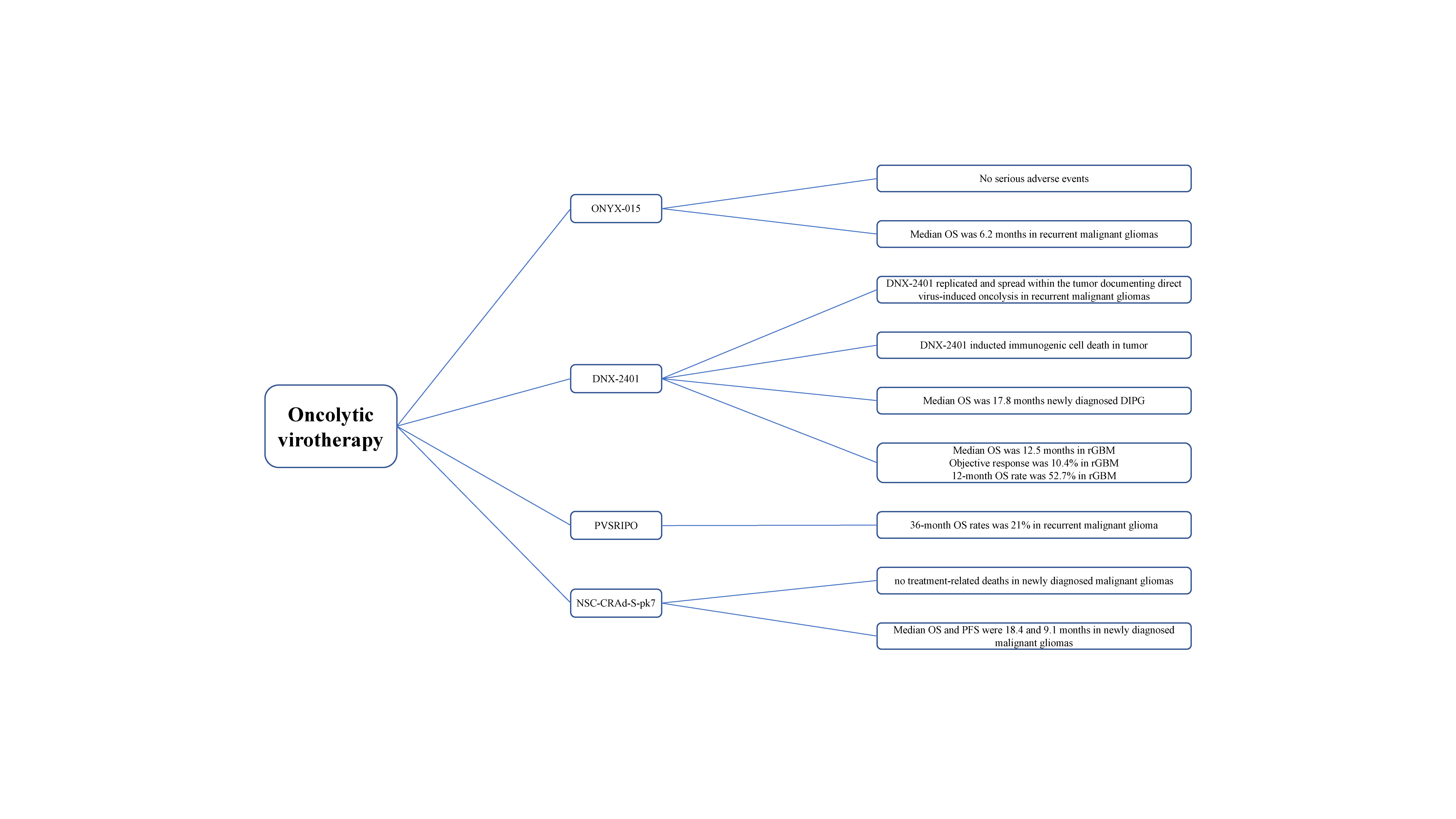

Supplement: Supplementary file 4 [file Image4.tif]
